# Phytochemicals as Potential Chemopreventive and Chemotherapeutic Agents for Emerging Human Papillomavirus–Driven Head and Neck Cancer: Current Evidence and Future Prospects

**DOI:** 10.3389/fphar.2021.699044

**Published:** 2021-07-20

**Authors:** Nikita Aggarwal, Joni Yadav, Suhail Chhakara, Divya Janjua, Tanya Tripathi, Apoorva Chaudhary, Arun Chhokar, Kulbhushan Thakur, Tejveer Singh, Alok Chandra Bharti

**Affiliations:** Molecular Oncology Laboratory, Department of Zoology, Faculty of Science, University of Delhi, Delhi, India

**Keywords:** head and neck cancer, human pappillomavirus, tobacco, smoking, phytochemicals, therapeutics, prevention

## Abstract

Head and neck cancer (HNC) usually arises from squamous cells of the upper aerodigestive tract that line the mucosal surface in the head and neck region. In India, HNC is common in males, and it is the sixth most common cancer globally. Conventionally, HNC attributes to the use of alcohol or chewing tobacco. Over the past four decades, portions of human papillomavirus (HPV)-positive HNC are increasing at an alarming rate. Identification based on the etiological factors and molecular signatures demonstrates that these neoplastic lesions belong to a distinct category that differs in pathological characteristics and therapeutic response. Slow development in HNC therapeutics has resulted in a low 5-year survival rate in the last two decades. Interestingly, HPV-positive HNC has shown better outcomes following conservative treatments and immunotherapies. This raises demand to have a pre-therapy assessment of HPV status to decide the treatment strategy. Moreover, there is no HPV-specific treatment for HPV-positive HNC patients. Accumulating evidence suggests that phytochemicals are promising leads against HNC and show potential as adjuvants to chemoradiotherapy in HNC. However, only a few of these phytochemicals target HPV. The aim of the present article was to collate data on various leading phytochemicals that have shown promising results in the prevention and treatment of HNC in general and HPV-driven HNC. The review explores the possibility of using these leads against HPV-positive tumors as some of the signaling pathways are common. The review also addresses various challenges in the field that prevent their use in clinical settings.

## Introduction

Head and neck cancer (HNC) constitutes a large group of cancers arising in different anatomical sites of the head and neck (HN) region, comprising the lip and oral cavity, larynx, nasopharynx, hypopharynx, oropharynx, nasal cavity, paranasal sinuses, and salivary glands. Over 90% of these neoplastic tissues are squamous cell carcinomas (SCCs). According to WHO estimates for 2019, HNC was one of the leading forms of cancer with 931,931 new cases, representing 4.9% of all cancer cases (Globocan, 2020). Lip and oral cavity cancer made up nearly 40% of the total HNC cases followed by the cancer of the larynx region. Mortality statistics reported by GLOBOCAN estimate 467,125 deaths due to head and neck cancers, representing 4.7% of all cancer deaths. Prevalence data for 2020 point to India as carrying the highest burden of head and neck cancer, with 143,242 cases, followed by China (100,871), the United States of America (51,533), and the Russian Federation (23,772). These numbers are alarming and draw attention to immediate action against this highly preventable cancer as the etiological agents are well known.

Tobacco use, excessive alcohol consumption, and lately, infection of human papillomavirus (HPV) are the established risk factors for HNC (Marur and Forastiere, 2016). The risk of HNC is 10-fold higher in smokers than that of HNC in nonsmokers (IARC, 2004). Although excessive alcohol consumption is an independent risk factor, it also increases the risk for smokers (Smith et al., 2004; Chaturvedi et al., 2015). In the past decade, however, there has been a shift in the anatomic distribution of HNC with an increasing occurrence of neoplastic lesions in the oropharynx (Sturgis and Cinciripini, 2007). A concordant decrease in smoking prevalence and increase in HPV prevalence has been noted, especially in the younger age-group. The review of clinical manifestations of HNC based on their anatomical, histological, and etiological factors revealed a dichotomy in treatment response (Aggarwal et al., 2020). The data strongly point toward existence of two distinct types of HNC, namely, one that is caused by tobacco and alcohol abuse or occupational exposure to various carcinogens, and the other which is caused by biological agents like infection of HPV and possibly the EBV. The evidence presented in the present manuscript suggests discrete differences among the two disease groups, with each requiring separate clinical management.

Most patients with HNC seek clinical intervention at advanced stages of the disease (Haddad and Shin, 2008). This trend is quite common in individuals of low socioeconomic status, who cannot afford expensive medical/surgical treatments. Despite a well-standardized treatment regimen, current therapy has a very low success rate as 30–60% of patients diagnosed develop recurrent locoregional cancer or second primary cancers even after complete remission (Hashim et al., 2019). A major underlying factor is onset of chemo/radioresistance and treatment failure (Nikolaou et al., 2018). Thus, better therapeutic options are needed to mitigate this challenge. Moreover, prevention of HNC at an early precancer/cancer stage could be another window of opportunity by which disease burden and mortality due to HNC could be reduced. Currently, prevention focuses on risk behavior reduction like cessation of tobacco and early diagnosis of the disease. However, there is an unmet need for new therapeutics that could effectively eliminate HNC cells, reduce the onset of chemo/radioresistance, and could prevent the progression of the disease.

Recently, there has been a renewed interest in phytochemicals and herbal derivatives with therapeutic correlates from traditional medicine in the treatment and prevention of HNC due to their safety, availability, efficacy, and low cost. A number of studies carried out to investigate screening of phytochemicals using different HNC cell lines, animal models, and clinical evaluation in patients showed potent anticancer activities in a small set of phytochemicals. However, very limited number of studies addressed the impact of these herbal derivatives on HPV infection and HPV-positive HNC. In this article, we have systematically reviewed the existing data on various phytochemicals demonstrating chemotherapeutic and chemopreventive activities against HNC with a special emphasis on phytochemicals/herbal derivatives that showed anticancer effects against HPV-positive HNC. Further, major deficiencies and actionable leads in this field have been highlighted.

## Head and Neck Cancer Spectrum

**FIGURE 1:**
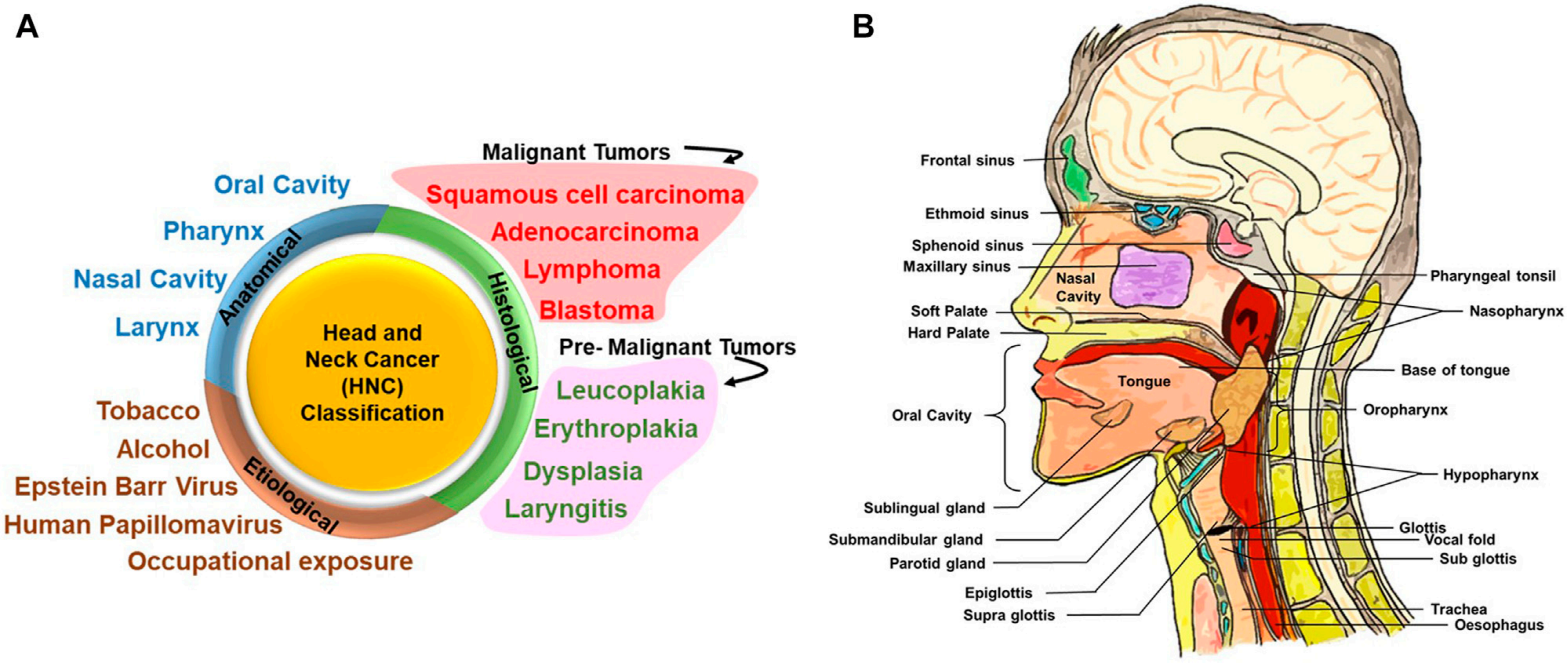
Head and neck cancer (HNC) classification and different anatomical sites involved. **(A)** HNC can be broadly classified on the basis of its anatomical site, histological origin, and etiological factors. Upon histological evaluation, tumors in the head and neck region can be broadly classified into malignant and premalignant lesions. Premalignant lesions that have been indicated here are not cancer but are precursors of malignant lesions. **(B)** Detailed anatomical architecture of the head and neck region illustrating the location of the oral cavity, nasal cavity, tongue, salivary glands (sublingual gland, submaxillary gland, and parotid gland), larynx and pharynx (including oropharynx, nasopharynx, and hypopharynx), and site of primary tumors.

HNC is a group of neoplastic diseases that can be broadly classified based on their anatomical site, histological origin, and etiological factors (Figure 1A).

### Anatomical Site–Specific Classification

Figure 1B illustrates the anatomic sites of the HN region. Broadly, the HN area is classified into four regions, namely, the oral cavity, pharynx, nasal cavity, and larynx. The oral cavity consists of the vestibule (the area between the teeth and mucosa of the lips and cheeks) and the oral cavity proper. The oral cavity proper is the interior region of the mouth: the region between the two dental arches and majorly occupied by the tongue (Akintoye and Mupparapu, 2020). Soft palate and hard palate separates the oral cavity from the nasal cavity.

Oral SCC (OSCC) arises from mucosal areas of the lips, front 2/3rd of the tongue, gums, internal lining of cheeks and lips, floor of mouth below the tongue, hard palate, and the area behind the wisdom teeth (Gartner, 1994), and constitutes a major proportion of cancers of the HN region. Globally, lip and oral cavity cancer prevalence is 34.7% among the overall cases of HNC. Lip and oral cavity cancer has the highest incidence in South-Central Asia (Globocan, 2020). The prevalence of lip and oral cavity cancer in the past 5 years is the highest in India, with a total burden of 300,413 cases. In the oral cavity proper, the tongue accounts for 40% of intraoral carcinomas (Neville and Day, 2002).

The pharynx is a channel located in the region of the neck midline. The pharynx is majorly classified into three regions: the nasopharynx (located posterior to nasal cavity), oropharynx (posterior end of oral cavity), and hypopharynx (behind the opening of larynx) (Albahout and Lopez, 2021). Globally, among HNC, the prevalence of the nasopharynx is 15.8%, which is the highest among three regions, followed by oropharynx 10.7%, which is higher than hypopharynx 5.5%. The incidence rate of the nasopharynx is high in Southeastern Asia, whereas the incidence rate of the hypopharynx is high in Central and Eastern Europe (Globocan, 2020). Incidence rates for the oropharynx are high in Europe, which is linked with alcohol consumption, tobacco smoking, and HPV. Incidence of HPV infection in the oropharyngeal region is rising at an alarming rate (Wierzbicka et al., 2021).

The nasal cavity is the upper most part of the respiratory tract. The nasal cavity is surrounded by four types of paranasal sinuses: frontal sinuses, sphenoid sinuses, paired maxillary sinuses, and ethmoid sinuses. Paranasal sinus malignancies are rare, accounting for less than 3–5% of the total HNC (Patel, 2017). The nasal cavity and paranasal sinuses disease burden are not covered by (Globocan, 2020) under HNC.

The internal space of the larynx is a pyramid shaped about 5 cm long, connecting the pharynx to the trachea and is a part of the respiratory system. According to Globocan (2020), the incidence of larynx cancer is highest in Central and Eastern Europe. Laryngeal cancer constitutes around 21.4% among HNC (Globocan, 2020).

Exocrine glands and salivary glands function to secrete saliva in the oral cavity. Three type of salivary glands are present: parotid gland (situated front of both ears), submandibular gland (posterior of the mandible), and sublingual gland (floor of the oral cavity) (Ghannam and Singh, 2021). In the salivary gland, majority tumors are benign, whereas malignant tumors are generally mucoepidermoid carcinoma and adenocarcinoma. Primary SCC is rare and aggressive in salivary glands, specifically in the parotid gland (Flynn et al., 1999). The incidence of the salivary gland cancer has been reported to be the highest in Middle Africa. Salivary gland cancer constitutes 6.6% of total HNC.

### Histological Classification

In the oral cavity, the mucosa is of masticatory, specialized, and mobile type. It covers around 25% of the oral cavity. In order to understand mechanical forces caused by mastication, it is covered by specialized, orthokeratinized, stratified squamous epithelium. Depending on the anatomic site, over 60% of the mucosa in the oral cavity is lined by the stratified squamous epithelium. The upper surface of the tongue is lined by specialized mucosa, with numerous lingual papillae (Winning and Townsend, 2000).

Histologically, the tumors of the HN region are classified as carcinoma, adenocarcinoma, lymphoma, and blastoma depending upon the tissue from where they are originating (Ologe et al., 2005). For instance, cancer originating in squamous cells in the HN region is collectively termed as HNSCC, and the one originating in salivary glands is of glandular origin and classified as an adenocarcinoma. The most common cancer affecting the HN region is epithelial carcinoma, which constitutes 80–90% of total cases, followed by lymphomas and blastomas accounting for the rest (Ologe et al., 2005; Gilyoma et al., 2015). Among carcinomas, squamous cell carcinoma constitutes 67.7% of total carcinoma cases, whereas other carcinomas like follicular carcinoma, adenocarcinoma, adenoid cystic carcinoma, clear cell carcinoma, mucoepidermoid carcinoma, and malignant melanoma cover the remaining carcinoma cases (Adeyemi et al., 2008).

Carcinomas mostly spread in the regions of the larynx, nasopharynx, and least in maxillofacial bones and oral cavity regions, whereas predominant anatomical sites for lymphomas were lymph nodes, followed by the maxillofacial bones. In contrast, sarcomas occurred most frequently in the maxillofacial bones, face/scalp, and the nose area (Adisa et al., 2011). The distribution of these tumors varies among the age-group of the patients. Most of the carcinomas are detected in the age-group of 45–64 years in contrast to sarcomas frequently occurring in the age-group of 25–44 years (Adeyemi et al., 2008; Adisa et al., 2011).

In the oral cavity, leukoplakia (white plaque) and its variants, erythroplakia (fiery red patch) and submucous fibrosis (most prevalent in India), are three conditions that are highly associated with the development of oral epithelial dysplasia (OED) and oral squamous cell carcinoma (OSCC). Malignant transformation rates of leukoplakia range from 8.9 to 17.5 percent (Silverman et al., 1984; Lind, 1987). The buccal mucosa had the highest incidence of leukoplakia, with 18% of lesions, but had the lowest rate of malignant transformation (3%). The tongue accounted for 16% of lesions but had the highest rate of transformation at 24% (Warnakulasuriya and Ariyawardana, 2016). Erythroplakia occurs mainly in the middle aged and the elderly and has the prevalence ranging from 0.02 to 1%. Soft palate, floor of the mouth, and buccal mucosa have their highest rate of incidence. The reason for etiopathogenesis has not been determined, but chewing tobacco and consuming alcohol have been implicated as factors for the development erythroplakia. The malignant transformation rate in erythroplakia is very high (14–50%) (Reichart and Philipsen, 2005). Oral submucous fibrosis is another chronic and potentially malignant disorder characterized by juxtaepithelial fibrosis of the oral cavity. This lesion has been reported to have a malignant transformation rate of 7–30%. Its incidence is highly associated with the chewing of betel quid containing areca nut (Ranganathan et al., 2004).

Dysplasia can be categorized as mild (architectural disturbance and cytological atypia in lower third of the epithelium), moderate (architectural disturbance and cytological atypia in middle third of the epithelium), and severe (architectural disturbance and cytological atypia in greater than two-third of the epithelium). This classification of dysplasia by the WHO is referred to as the gold standard for histological diagnosis of oral potentially malignant disorders (OPMDs). The WHO defines OPMDs as “clinical presentation that carry a risk of cancer development in the oral cavity, whether in a clinically definable precursor lesion or in clinically normal mucosa” (Muller, 2018). Epithelial dysplasia, an important precursor of malignant transformation in the HN region, can be defined as a change in morphological characteristics of the epithelium, including architectural and cytotoxic changes and loss of differentiation of keratinocytes toward the surface. It involves replacement of a part or the entire epithelium by cells showing cellular atypia (Tilakaratne et al., 2019; Wils et al., 2020).

The stratified squamous epithelium lines the pharynx to protect it from mechanical stress. The pharynx and larynx both are lined with the ciliated pseudostratified columnar epithelium with goblet cells. A study suggests that lesions such as erythroplakia at high-risk sites in the oropharynx should be considered as invasive carcinoma or carcinoma *in situ* at high-risk sites unless a biopsy proves otherwise (Mashberg and Samit, 1995). However, the vocal cords are lined with the stratified squamous epithelium (Stiblar-Martincic, 1997). Although there is no consensus, premalignant lesions of the larynx are usually classified as chronic laryngitis, erythroplakia, leukoplakia, and erythroleukoplakia (Gale et al., 2009). In the premalignant and malignant lesions of the larynx, severe dysplasia and carcinoma *in situ* occur at the rate of 10–20% (Hellquist et al., 1982). The nasal mucous membrane is lined with the sensory epithelium with olfactory cells and the respiratory epithelium. The mucosa is rich in mucus-producing goblet cell. Nasal drainage is facilitated by the ciliated epithelium. Premalignant lesions of paranasal sinuses differ from other lesions of the HN region and are present as inverted papillomas. This cancer goes undiagnosed before the onset of symptoms. Malignant tumors of paranasal sinus are diagnosed at stages T3–T4 in two-thirds of cases. Additionally, in paranasal cancer, 10% of total SCCs and 4% of all adenocarcinomas have some degree of cervical lymph node involvement (Jegoux et al., 2013). Salivary glands constitute three cell types, namely, acinar cells, myoepithelial cells, and ductal cells (Brazen and Dyer, 2020). In the parotid gland, 70% of the tumors detected are benign. In the submandibular gland, adenoid cystic carcinoma is the common malignancy (16%). Sublingual gland tumors are rare but have the highest frequency of malignancy, ranging from 70 to 90% (Carlson and Schlieve, 2019).

### Classification Based on Etiological Type

**Tobacco-associated HNC:** Association of tobacco and alcohol use with the onset of HNC is well established (IARC, 2004). Tobacco use is the leading cause of preventable death in the world. Tobacco smoking alone is the leading cause of cancer and cancer-related deaths worldwide. Nearly 85% of HNC are linked with tobacco use. Within the HN region, it has been conclusively shown to directly cause oral cavity, laryngeal, and pharyngeal cancer (Centers for Disease Control and Prevention, 2004). The International Agency for Research on *Cancer* (IARC) has classified carcinogens in groups, group 1: tobacco smoking, secondhand smoking, and smokeless tobacco for HNCs, which are sufficient for evident carcinogenicity in human (IARC, 2004). In developed countries, most inhaled or “mainstream” tobacco smoke comes from the use of manufactured cigarettes. Cigarettes burn at very high temperature and produce smoke that includes toxins and carcinogens. Similar drawbacks are with cigars, pipes, and water pipes (IARC, 2004).

Tobacco smoke contains a variety of group 1 carcinogens, namely, arsenic and benzene, but research is more focused on tobacco-specific N-nitrosamines, especially N-nitrosonornicotine and 4-(N-nitrosomethylamino)-1-(3-pyridyl)-1-butanone, as they are established carcinogens. In HNC of HNSCC type, the latter one is more associated with increasing the risk of cancer development (Oreggia et al., 1991). Tar is another compound which is linked with an increased risk of HNC (Franceschi et al., 1992).

Studies have shown that development of HNC is strongly related with dose-dependent tobacco smoking but can also occur with low daily usage (Berthiller et al., 2016). Moreover, the duration of exposure also significantly affects the risk of HNC. The risk of daily smoking for more than 30 years was found to be more carcinogenic (Cohen et al., 2018).

**Alcohol-associated HNC:** HNC is also associated with alcohol abuse. Studies suggest that alcohol consumption and cigarette smoking are differentially associated with the risk of HNSCC subtypes (Bagnardi et al., 2001). A large prospective study has confirmed that alcohol consumption is strongly linked to HNSCC (Freedman et al., 2007). Among all, oropharyngeal SCC (OPSCC) is the most associated, while laryngeal SCC (LSCC) is the least associated with heavy alcohol consumption (Zeka et al., 2003; Lubin et al., 2009; Toporcov et al., 2015). Clinically, there is no distinction between alcohol- and tobacco-associated HNC.

**Occupational exposure**–**associated HNC:** Apart from smoking of tobacco products, occupational exposure to dusts from wood, textiles, leather industries, flour, nickel, chromium, fumes from rubbing alcohol (also called isopropyl alcohol), radium, glue, formaldehyde as well as solvent fumes used in furniture and shoe production, and asbestos are the main risk factors for sinonasal carcinomas. Hypopharygeal and laryngeal carcinoma are associated with the use of coal for heating or cooking (IARC, 2012). These tumors have an aggressive clinical behavior and resemble tobacco-associated tumors in progression and therapeutic response.

**Epstein**–**Barr virus**–**associated HNC:** The etiology and natural history of nasopharyngeal SCC (NPSCC) is closely linked to that of Epstein–Barr virus (EBV) infection. This neoplasm is an uncommon disease with very low prevalence in most countries (Wei and Sham, 2005). Although EBV infection is pervasive, NPSCC incidence differs considerably around the world (Chang and Adami, 2006). In most geographical regions where NPSCC is endemic, the onset of EBV infection occurs at an early age. The estimated latency period of this virus is around a decade, so other factors also contribute for NPSCC development. Evidences indicate that this cancer is predominant in individuals of Southeast Asian descent due to genetic differences (Chang and Adami, 2006; Bei et al., 2016; Liu et al., 2017).

**HPV-associated HNC:** HPV is a DNA virus with oncogenic potential associated with over a dozen genotypes referred to as high-risk HPV. Persistent HPV infection is chiefly associated with the development of anogenital and cervical carcinomas. HPV16 and HPV18 genotypes are the most prevalent carcinogenic types and act *via* action of two major oncogenes, E6 and E7. These oncogenes target cell cycle and promote tumor growth by targeting and downregulating p53 and pRb, respectively. Many molecular and epidemiological studies support association of HPV with HNC, especially with OPSCC (Franceschi et al., 1996). Over the last 125 years, observations speculating the presence of a virus transmitting oral tumors have matured and led to the identification of a subset of HNC with distinct clinical presentation that show an early onset (Table 1). Approximately 35% of all HNC and 77% of tonsillar cancers harbor HPV, with greater than 60% of cases being the HPV16 subtype (McKaig et al., 1998). A significant variation in HPV prevalence in HNC types is recorded within different studies and from different geographical regions (Gillison et al., 2015).

**TABLE 1:** Major historical milestone events in the description of HPV infection in the head and neck region (adapted from Syrjanen et al. (2017)).

Year	Milestones	References
1891, 1896	First speculation of contagious nature of cutaneous warts	Payne (1891), Jadassohn (1896)
1901	Contagious transmission of condyloma warts in the tongue after oral sex described	Heidingsfield (1901)
1907	Viral etiology of oral lesions	Ciuffo (1907)
1923	Association of human wart virus with laryngeal warts established	Ullman (1923)
1943	Oral papillomatosis as a viral disease was established in rabbits	Parsons and Kidd (1943)
1948, 1956	Reporting of koilocytotic atypia in laryngeal papilloma	Ayre and Ayre (1949), Ishiji et al. (1992)
1978	Epithelial atypia in laryngeal papilloma reported	Quick et al. (1978)
1973	Identification of HPV in laryngeal papilloma	Boyle et al. (1973)
1974–75	Detected virus-specific DNA in human tumors	zur Hausen et al. (1975)
1976–77	HPV association with koilocytotic atypia established as a sign of HPV infection	Meisels and Fortin (1976), Purola and Savia (1977)
1978	Development of noncommercial antiserum against HPVs	Pyrhonen and Neuvonen (1978)
1980	HPV 6 was isolated from condyloma acuminata	Gissmann and zur Hausen (1980)
1982	Expression of HPV structural proteins in laryngeal carcinoma	Syrjanen et al. (1992)
1982	HP 11 was detected in laryngeal papilloma	Gissmann et al. (1982)
1982	HPV detection in benign and malignant oral SCC	Jenson et al. (1982), Syrjanen et al. (1983a)
1983	An extensive squamous cell papilloma of the nasal cavity and also filling the entire left maxillary sinus is reported	Syrjanen et al. (1983b)
1983	Morphologic and immunohistochemically features indicate HPV infection in OSCC	Syrjanen et al. (1983a)
1987	HPV DNA in benign and malignant sinonasal lesions	Syrjanen et al. (1987)
1989	Detection of HPV DNA in human oral tissue biopsies and cultures	Maitland et al. (1989)
1989	HPV16 DNA detection in tonsillar carcinoma	Brandsma and Abramson (1989)
1992	Success in preparation of virus-like particles (VLPs), namely, BPV1 and HPV16, that established HPV serology and vaccination	Kirnbauer et al. (1992)
1992	First report showing the presence of transcriptionally active and integrated HPV infection with expression of E6/E7 mRNAs in tonsilar cancer	Snijders et al. (1992)
1992	HPV16/18 DNA in nasopharyngeal carcinoma	Dickens et al. (1992)
2004	Papillomaviruses recognized as a taxonomic family of their own	De Villiers et al. (2004)
2005	Differential expression and activity of transcription factors in HPV-positive oral cancers	Mishra et al. (2006)
2008	Halard zur Hausen was awarded with Nobel prize in physiology or medicine	—

**FIGURE 2:**
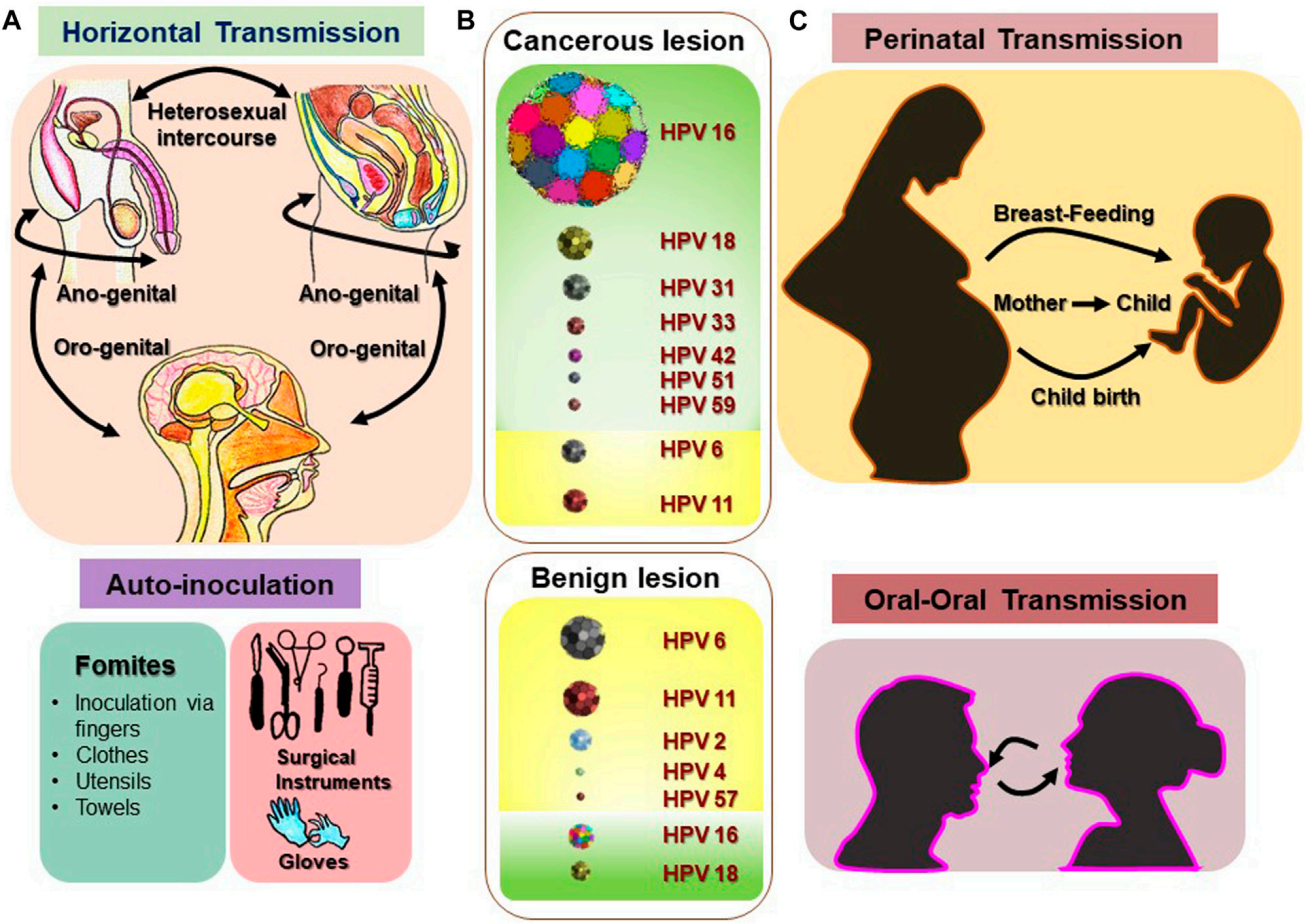
Various routes and sites of HPV transmission. HPV is a sexually transmitted infection that can be received through horizontal transmission (heterosexual intercourse, anogenital, and oro-genital) and in some rare cases through various fomites, and inoculation *via* fingers, clothes, utensils, towels, and surgical instruments. **(A)** Vertical transmission (perinatal transmission: mother to baby) and oral–oral transmission of HPV could be the main source of nonsexual transmission of HPV in oral sites. **(C)** Most prevalent high-risk and low-risk HPV types reported in benign and malignant lesions of the HN region are depicted with representative figures where the relative frequency of the each type is indicated with corresponding sizes. **(B)** [Disclaimer: Pictures used to make composite diagram and to represent HPV types are derived from different internet sources and does not claim to be the original representation of the indicated HPV type] (Sarkola et al., 2008; Chaturvedi et al., 2015; Fu et al., 2015; Visalli et al., 2016; Louvanto et al., 2017; Sabeena et al., 2017; Syrjanen, 2018; Houlihan et al., 2019).

Finding HPV in the HN region is paradoxical. However, a sexual mode of transmission has been suggested. Due to muco-epithelial tropicity of these viruses, if the virus gets access to these tissues *via* opportunistic contact with infected genital organs, it can result in the establishment of HPV infection in the HN region (Figure 2). Patients with other HPV-associated neoplasms or premalignant conditions are presumed to be at a higher risk of HNC development. Among spouses, women having a history of cervical dysplasia showed higher incidence of HPV-related oropharyngeal cancer (Hemminki et al., 2000). Patients with a history of anogenital cancer have shown a higher risk of tonsillar cancer (Frisch and Biggar, 1999). These HPV-positive cancers are primarily SCCs in their histological manifestations.

### Recognition of Human Papillomavirus–Positive HNSCC as a Distinct Human Papillomavirus–Driven Subtype

Data emerged in last 2 decades strongly support the recognition of HPV-positive HNSCC as a distinct disease with a well-defined clinical and molecular pattern and unique risk factors (Table 2). These HPV-positive tumors were reported in early stage (Pintos et al., 1999; Smith et al., 2004; Hammarstedt et al., 2006), well differentiated histology (Pintos et al., 1999; Gupta et al., 2015), basaloid morphology (Gillison et al., 2000), larger tumors (Gletsou et al., 2018), and either no lymph node involvement (Pintos et al., 1999) or with cystic cervical lymph node positivity (Goldenberg et al., 2008). These tumors showed low risk of second primary malignant neoplasm (Adjei Boakye et al., 2018) with a better overall and disease-free survival (Ragin and Taioli, 2007; Fakhry et al., 2008; Ang et al., 2010; Rischin et al., 2010; Posner et al., 2011; Fakhry et al., 2014). Irrespective of the tissue subtype involved, HPV positivity in HNSCC emerged as a strong biomarker associated with better prognosis (Gillison et al., 2000; Wookey et al., 2019).

**TABLE 2:** Representative studies demonstrating the existence of HPV-positive HNC as a distinct disease group.

Study (year)	Sample size and HPV positivity	Study design	Anatomical sites examined	HPV-positive HNSCC	HPV-negative HNSCC
Pintos et al. (1999)	Archival specimens of UADT (*n* = 101), HPV positivity: 16.8%	Cross-sectional study	Pharynx, buccal, larynx	Gender bias [M:F::14:3 (4.7)]	Gender [M:F::66:18 (3.7)]
				Younger age [<60:>60 years::12:8 (1.5)]	Age [<60:>60 years::30:54 (0.55)]
				Higher proportion of WDSCC [6/17 (0.35)]	WDSCC [14/84 (0.17)]
				Early stage [T1-2:T3-4::11:6 (1.8)]	Stage [T1-2: T3-4::41:43 (0.95)]
				Without lymph node metastasis [2/17 (0.12)]	Lymph node metastasis [29/84 (0.35)]
Gillison et al. (2000)	Fresh tissues (*n* = 253), HPV positivity: 22%	Prospective analysis of tissues with patient follow-up and association with history	All sites of the HN region	HPV16 associated, viral integration, poor tumor grade (OR-2.4)	Moderate-to-heavy drinkers (OR- 5.88)
				Over-representation in oropharynx	Smokers (OR- 6.25)
				Basaloid morphology (OR- 18.7)	TP53 mutations detected (OR- 16.7)
				Better DFS (HR- 0.26)	Age at diagnosis >60 years
				Better prognosis (59% risk reduction)	
Van Houten et al. (2001)	Fresh specimens of UADT (*n* = 84)	Prospective analysis	All sites of the HN region	p53 wild type, non-mutated in E6 positive tumor (9/9)	Frequent p53 mutations [40/64 (62.5%)]
	HPV positivity: 23.8%			p53 mutations only in HPV E6RNA negative tumors [4/11 (36.4%)]	
Mork et al. (2001)	Serum from cohort studies (cases = 292; controls = 1,568)	Case–control retrospective study	All sites of the HN region	Seropositivity for HPV16–35/292 (12%) against control group—102/1,568 (7%)	Reference
	HPV positivity: 12%				
Smith et al. (2004)	Patient biopsy (*n* = 193)	Prospective analysis	All sites of the HN region	Younger age (<55:>55 years; OR-3.4)	Reference
	HPV positivity: 20%			More lifetime sex partners (OR-3.8), practiced oral-genital sex (OR-4.3), or oral–anal sex (OR-19.5)	
Mishra, et al. (2006)	Patient biopsy (*n* = 66)	Prospective analysis	All sites of the HN region	Selective participation of p65 subunit in the NF-κB complex	Constitutively active NF-κB complex with p50 homodimer
Mishra et al. (2006)	HPV positivity: 27%				
Hammarstedt et al. (2006)	Archival specimens (*n* = 203)	Retrospective study of cases b/w 1970–2002	Tonsils	Younger patients [<60:>60 years::58:41 (1.41)]	Age [<60:>60 years::30:74 (0.41)]
	HPV positivity: 49%				
Ragin and Taioli (2007)	Pooled analysis (*n* = 1747)	Meta-analysis	Oral cavity, oropharynx	Lower risk of dying (HR-0.85)	Reference
	HPV positivity: 27.7%			Lower risk of recurrence (HR-0.62)DFS (HR: 0.51)	
Fakhry et al. (2008)	Fresh tissues (*n* = 96 patients)	Prospective clinical trial controlled for known factors of prognostic values	Oropharynx, larynx	Higher response after induction chemotherapy (82%) and chemoradiation (84%)	Moderate response after induction chemotherapy (55%) and chemoradiation (57%)
				Increased 2-years survival (95%) with lower risk of progression and death	
	—			Lower risk of dying (HR-0.36)	—
				Lower risk of progression (HR-0.27)	
Chaturvedi et al. (2008)	SEER (1973–2004) (*n* = 45,769)	Cohort analysis for investigation of survival of OSCC patients	Oral cavity	Mean ages at diagnosis-61.0 years	Mean ages at diagnosis-63.8 years
	HPV positivity: 38.5%			APC in incidence (1973–2004) - 0.80 Showed increased	APC in incidence (1973–2004)–(−)1.85
				2-year survival from 9.9 to 18.6%	Showed 2-year survival from 5.6 to 9.9%
Gillison et al. (2008)	Newly diagnosed HNSCC patient (*n* = 240) and 322 controls [HPV(16) positivity: 38.3%]	Case–control study to compare risk factors in HPV-positive vs HPV-negative tumors	Oral cavity, paranasal sinus, pharynx, larynx	Gender bias [M:F::78:14 (5.6)] association increased with the increasing number of oral sex partners, with increasing intensity (joints per month), duration (in years), and cumulative joint-years of marijuana use	Gender bias [M:F::111:37 (3.0)]
					Associated with tobacco smoking, alcohol drinking, and poor oral hygiene
					Not associated with sexual behavior or marijuana use
Golderberg et al. (2008)	FFPE (*n* = 84) [HPV(16) positivity: 87%]	Retrospective review of patients undergoing neck dissection between 2002 and 2004	Oropharynx, oral cavity, larynx, hypopharynx	Related with cystic cervical lymph node	Associated with solid nodal metastasis
Ang et al. (2010)	Patients (*n* = 323)	Retrospective analysis for tumor HPV status and survival among patients	Oropharynx	3-year rate of survival (82.4%)	3-year rate of survival (57.1%)
				3-year rates of PFS (73.7%)	
				Reduction in the risk of death (58%)	—
				Reduction in the risk of relapse or death (51%)	
Rischin et al. (2010)	Stage III and IV patients (*n* = 172)	Retrospective study	Oropharynx	Lower T and higher N categories and better ECOG performance status in p16 positive. 2-year overall survival [91% (HR-0.36)]. 2-year failure-free survival in p16 positive [87% (HR-0.39)]	2-year overall survival (74%). 2-year failure-free survival (72%)
	HPV positivity: 53.5%; p16 positivity^a^–59.3%				
Chaturvedi et al. (2011)	Archival tissue from year 1988 to 2004 (*n* = 271)	Retrospective time period study	Oropharynx	Median survival (131 months)	Median survival (20 months). Population-level incidence declined (50%; 2.0–1.0 per 100,000)
				Increased prevalence from 1984 to 1989 (16.3%) to 2000 to 2004 (71.7%)	
				Population-level incidence increased (225%; from 0.8 per 100,000 to 2.6 per 100,000)	
Posner et al. (2011)	Patients (*n* = 111)	Retrospective study to evaluate OS, PFS, and HPV	Oropharynx	Median age: 54 years	Median age: 58 years
				T1/T2 primary: 49%	T1/T2 primary: 20%
	—			5-year PFS: 78%	5-year PFS: 28%
				5-year OS: 82%	5-year OS: 35%
De Martel et al. (2012)	GLOBOCAN data 2008 (sample size not described)	Synthetic analysis of HPV PCR positivity in tumor tissue with HPV E6 or E7 expression	Oropharynx	Geographical variations (north America: 56%, northern and western Europe: 39%, eastern Europe: 38%; southern Europe: 17%, Australia–45%, Japan: 52%, rest of world: 13%	Not assessed
Ndiaye et al. (2014)	Patients (*n* = 12,163) [overall HPV positivity: 31.54%; for oropharynx: 45.8%, for larynx (including hypopharynx): 22·1%, and for oral cavity: 24·2%]	Meta-analysis of 148 studies	Oropharynx, larynx, oral cavity	p16INK4a positivity in HPV-positive oropharyngeal cancer cases: 86·7% and E6/E7 mRNA positivity: 86·9%	Reference
				HPV attributable fraction in oropharyngeal cancer defined by expression of positive cases of E6/E7 mRNA was estimated as 39·8% and of p16INK4a was 39·7%	
Fakhry et al. (2014)	Patients (*n* = 181)	Retrospective evaluation of OS	Oropharynx	Improved 2-year OS in p16 positive patients (54.6%; median: 2.6 years)	OS in p16-negative patients (27.6%; median: 0.8 years)
	p16 positivity^a^-58%				
Vermorken et al. (2014)	Patient samples-FFPE (*n* = 416)	Retrospective analysis of R/M HNSCC	All sites of the HN region	Better OS for HPV+/p16+. CT + cetuximab (median month-12.6). CT (median month-7.1)	OS for HPV-/p16-CT + cetuximab (median month-9.6). CT (median month-6.7)
	HPV positivity: 6%				
The Cancer Genome Atlas Network (2015)	Tumor tissues (*n* = 279)	Cohort study	Oral cavity oropharynx, larynx	Helicase domain mutations of the oncogene PIK3CA. Novel alterations involving loss of TRAF3. Amplification of the cell cycle gene E2F1	Near universal loss-of-function TP53 mutations and CDKN2A with frequent copy number alterations including a novel amplification of 11q22
	HPV positivity: 12.9%				
Gupta et al. (2015), Gupta et al. (2018)	Fresh biopsies (*n* = 50) [HPV(16) positivity: 28%]	Prospective study	Tongue	Well differentiated tongue carcinomas (78.5%)	Poorly differentiated carcinomas (72.2%)
				Higher expression and DNA binding activity of AP-1 and NF-κB with c-fos and Fra-2; and p50 and c-rel as the major binding partners forming the functional AP-1 and NF-κB complex, and selective participation of p65	Low expression and DNA binding activity of AP-1 with c-Jun as the major binding partners forming the functional AP-1 complex
				Induced expression of p65 and p27 leading to well differentiation and better prognosis	Participation of c-Rel with p50 that in crosstalk with AP-1/Fra-2 leading to poor differentiation and aggressive tumorigenesis
Gaykalova et al. (2015)	Tissues from HNSCC patients (*n* = 195) and noncancer-affected patients (*n* = 63) [discovery- HPV(16) positivity: 29.5%]	Cohort study	All sites of the HN region	Described 5 top-scoring pair biomarkers from STATs, NF-κB and AP1 pathways that distinguished HPV + HNSCC based on TF activity	High expression of CCND1, CEBPD, ICAM1, IRF1, JAG1, JAK3, and NOS3
Verma et al. (2017)	Fresh biopsies and FFPE tissues (*n* = 135) [HPV(16) positivity: 23%]	Prospective and archival study	Oral cavity oropharynx	Direct correlation with tissue immunopositivity for JunB and p65, whereas pSTAT3 were inversely correlated	Presence of STAT3/pSTAT3 with NF-κB irrespective immunopositivity for AP-1 members
				Low pEGFR^Y1092^ status	High pEGFR^Y1092^ status
Gletsou et al. (2018)	Patient samples-FFPE (*n* = 28)	Analytical study	Oropharynx	Bigger	Comparatively smaller
	HPV positivity: 10.7%			Tumor diameter of 3.7 ± 1.5 cm, volume of 9.5 ± 5.8 cm^3^	Tumor diameter of 2.7 ± 0.6 cm, volume of 5.4 ± 2.7 cm^3^
Adjei Boakya et al. (2018)	Patient samples (*n* = 109,512) from SEER	Cohort study	All sites of the HN region	Low risk of second primary malignant neoplasms	High risk of second primary malignant neoplasms
	HPV positivity: 38.1%				
Abdel-Rahman (2020), (Abdel-Rahman, 2020)	Patient records (*n* = 1,157) from SEER	Cohort analysis for investigation of survival of hypopharyngeal carcinoma patients	Hypopharynx	OS (HR: 1.76)	Reference
	HPV positivity: 24%			Better OS with regional and distance disease	
				Head and neck cancer–specific survival (HR: 1.54)	

a p16 positivity was taken as surrogate marker for (transcriptionally active) HPV positivity.

Abbreviations: AP1, activator protein 1; APC, annual percentage change; CT, chemotherapy; DFS, disease-free survival; DNA, deoxyribose nucleic acid; ECOG, Eastern Cooperative Oncology Group; EGFR, epidermal growth factor receptor; FFPE, formalin fixed paraffin embedded; HPV, human papillomavirus; HR, hazard ratio; HN, head and neck; HNSCC, head and neck squamous cell carcinoma; NF-κB, nuclear factor-kappa B; OS, overall survival; OR, odds ratio; OSCC, oral squamous cell carcinoma; OPSCC, oropharyngeal squamous cell carcinoma; PIK3CA, phosphatidylinositol-4,5-bisphosphate 3-kinase catalytic subunit alpha; PCR, polymerase chain reaction; PFS, progression-free survival; R/M, recurrent and/or metastatic; STAT3, signal transducer and activator of transcription 3; SEER, surveillance, epidemiology, and end result program registries; TRAF3, TNF receptor-associated factor 3; UADT, upper aerodigestive tract; WDSCC, well-differentiated squamous cell carcinoma.

HNSCC is overrepresented in males (Pintos et al., 1999; Gillison et al., 2008). The gender bias increases further in HPV-positive tumors (Pintos et al., 1999). Gender-specific data derived from HPV-positive oropharyngeal cancer (OPC) patients showed a higher risk of premalignant lesions in men (Ryerson et al., 2008). These observations are indicative of a tumor-promoting role of either male-specific hormones leading to differences in clearance of HPV infections due to the endocrine-immune interactions (Klein, 2000), or a distinct cellular environment in oral mucosal cells of men that promote transcriptional activation of viral oncogenes and HPV-mediated HNC. An increased anal HPV16/18 prevalence has been noticed, which correlated with high free testosterone levels in men having sex with men (Hsu et al., 2015).

Time trend studies carried out in different cohorts and registries particularly in North America and Europe revealed an interesting disease dynamics among all the HN sites (Chaturvedi et al., 2008; Chaturvedi et al., 2011). OPC showed a characteristic change in incidence (Hammarstedt et al., 2006; Mehanna et al., 2013). During the 30 year period, HPV-negative OPC declined steeply with a simultaneous and more prominent emergence of HPV-positive OPC (Chaturvedi et al., 2011). HPV-negative OPC and non-OPC that included all other HN sites are HPV-unrelated and traditionally linked to smoking and alcohol abuse. On the contrary, the studies showed a definitive and strong link of HPV-positive tumors with the oral–genital sexual contact (Gillison et al., 2008). The HPV-positive HNSCC shows large variations in prevalence among different geographical regions (De Martel et al., 2019) and may be associated with prevailing sociocultural and sexual practices, whereas genetic predispositions that may also play a sizable role in this phenomenon cannot be ruled out. In line with these observations, a higher incidence of HPV-positive tumors in Hispanic population has been reported (Gillison et al., 2008).

Early studies repeatedly pointed to a lower median age of HPV-positive HNSCC (Chaturvedi et al., 2008; Posner et al., 2011). However, a recent study demonstrated increased HPV positivity even in older age-group (Windon et al., 2018), thus indicating that early onset of HNSCC was merely circumstantial. Reviewing the factors contributing to the changing pattern of HNSCC over last 50 years revealed a major shift in societal practices with respect to depiction of sexuality (Syrjanen et al., 1982). Surprisingly, in 1969, Denmark legitimized display of explicit content, which was followed by the Netherlands and Sweden, and by 1972, the United States observed a peak in the films displaying oral sexual acts. Therefore, the shift in the HNSCC from HPV-negative to HPV-positive tumors observed in the western population is possibly associated with two independent phenomena that occurred simultaneously. First, establishment of tobacco’s carcinogenic potential (Vizcaino et al., 2015) and consequent implementation of anti-tobacco policies; and second, display of oral sex on motion pictures that promoted indulgence in high-risk behavior leading to increased exposure of oral mucosa to genital HPV infections. Treatment efficacy can be maintained by evaluating the HPV-positivity in OPSCC; as they have better prognosis, they can be treated with less aggressive treatment to avoid serious side effects to reduce treatment-associated toxicities in relatively younger patients (Boscolo-Rizzo et al., 2016).

### Molecular Signatures of Human Papillomavirus–Positive Head and Neck Cancer

During first 2 decades, research was emphasized on the detection of HPV and its distribution in the HN region. Subsequent studies revealed a series of distinctive molecular features in HPV-positive HNC (Table 3). In HPV-positive tumors, wild-type p53 was functionally active and was downregulated by E6 oncoprotein. Reduced p53 transcript was associated with the activation of many oncongenic pathway genes, which contributes to genetic instability in the development of cancer (van Houten et al., 2001; Licitra et al., 2006). HPV-positive HNC lesions show characteristically high expression of p16^INK4a^, which serves as a surrogate marker for HPV (Licitra et al., 2006). In contrast, HPV-negative tumors showed inactivating p53 and p16^INK4a^ mutation in HNSCC.

**TABLE 3:** Representative studies showing specific molecular differences in HPV negative vs HPV positive HNC.

Study (Year)	Sample size and HPV positivity	Study design	Anatomical sites examined	HPV-positive HNSCC	HPV-negative HNSCC
Van Houtenet al. (2001)	Fresh specimens of UADT (*n* = 84)	Prospective analysis	All sites of H&N region	p53 wild type, non-mutated in E6 positive tumor	Frequent p53 mutations (62.5%)
	(HPV positivity- 23.8%)			p53 mutations only in HPV E6RNA negative tumors (36.45%)	
Licitra et al. (2006)	Patient samples (*n* = 90). HPV positivity- 55.6%	Retrospective study	Oropharynx	p16 expression as surrogate marker	Homozygous deletion in p16^INK4a^–47%
				Normal p16^INK4a^ gene-100%	Normal p16INK4a gene-52.5%
				P16 immunophenotype-100%	P^16^ immunophenotype-21%
				Integration of HPV16 DNA-17%	TP53 mutation-48%
				TP53 mutation-39%	—
Zhang et al. (2013)	Patient samples (*n* = 325)	Case control study	Oropharynx, oral cavity	Short telomere length in PBLs—increased risk of OPC.	Reference
	HPV positivity- (OPC-46.27%)			No association was observed between telomere length in PBLs and risk of OCC.	
	HPV positivity- (OCC-9.5%)			—	
Chung et al. (2015)	Data from TCGA cohort	Observational study	All sites of H&N region	Genomic alterations of PIK3CA and PTEN genes	Genomic alterations of CDKN2A/B and TP53 genes
	Patients FFPE tissue (*n* = 252)			Altered pathways- PI3K pathway	Altered pathways- DNA repair p53 and cell cycle pathways
Seiwert et al. (2015)	Patients (*n* = 120)	Cohort study	All sites of H&N region	Unique mutational spectrum- mutation in DDX3X, FGFR2/3 and aberrations in PIK3CA, KRAS, MLL2/3, and NOTCH1 genes	Mutational spectrum- mutation in TP53, CDKN2A, MLL2, CUL3, NSD1, PIK3CA, and NOTCH genes
	(HPV positivity-42.5%)			Somatic aberrations in DNA-repair genes (BRCA1/2, fanconi anemia genes, and ATM)	
Pollock et al. (2015) (Pollock et al., 2015)	FFPE pretreatment tissue samples of HNSCC (n = 88)	Retrospective cohort based study	All sites of H&N region	Elevated expression of total HER2, total HER3, HER2:HER3 heterodimers, and the HER3:PI3K complex	Elevated expression of total EGFR (HER1)
Masterson et al. (2015)	Prospective cohort (*n* = 24)	Cohort based study	Oropharynx	Retrospective cohort- increased expression of CDKN2A transcript	Reference
	Retrospective cohort (*n* = 27)			Prospective cohort- increased expression of SYCP2 transcript	
	(HPV positivity-80.4%)			—	
Partlova et al. (2015) (Partlova et al., 2015)	Prospective study (*n* = 54)	Prospective study	All sites of H&N region	High infiltration rate of CD8^+^ IFNγ^+^ T lymphocytes, Tc17 lymphocytes, naïve CD4^+^ T lymphocytes and myeloid DCs. Production of high level of chemokines CXCL9, CXCL10, CXCL12, CXCL17 and CXCL21. Lower expression of Cox-2 mRNA. Higher expression of PD1 mRNA	Reference
	(HPV positivity-54.5%)				
Verma et al. (2017)	Fresh biopsies and FFPE tissues (*n* = 135)	Prospective and archival study	Oral cavity, oropharynx	Low expression of STAT3 and pSTAT3	Moderate or strong expression of STAT3 and pSTAT3
	(HPV positivity- 23%)			Strong or moderate expression of NF-κB and p65	No expression of NF-κB
	—			Marked expression of AP-1 family members	Low expression of p65 irrespective of presence or absence family member AP-1
Hajek et al. (2017)	TCGA (*n* = 279)	Cohort study	All sites of H&N region	Mutations in TRAF3 or CYLD genes activate NF-κB signaling	Gene alterations in TRAF3 (2%)
	(HPV-positivity 12.9%)				
Koneva et al. (2018)	HPV^+^ UM tumors (*n* = 18)	Prospective and archival study	Oropharynx, oral cavity, larynx, hypopharynx	Recurrent integration of CD274, FLJ37453, KLF12, RAD51B, and TTC6 genes	Reference
	HPV^+^ TCGA tumors (*n* = 66)			Integrated genes interact with Tp63, ETS, and/or FOX1A	
Ren et al. (2018)	Discovery cohort (*n* = 75)	Cohort study	Oropharynx	Hypermethylation of ATP5EP2, OR6S1, ZNF439, VSTM2B, ZNF137P, ZNF773 DMRs	Hypomethylation of ATP5EP2, OR6S1, ZNF439, VSTM2B, ZNF137P, ZNF773 DMRs
	(HPV positivity-66.675)			—	
	Validation cohort (*n* = 46)			—	
	(HPV positivity-52.17%)				
Gerle et al. (2018)	HPV positive cell line (UDSCC-2)	*In-vitro* study	Hypopharynx and tongue	Significant increase in asmase activity after irradiation and more sensitive to cisplatin treatment	Not significant increase in asmase activity after irradiation
Brand et al. (2018)	HPV positive cell lines (93-vu-147T, UPCI-SCC152, UPCI-SCC90, UM-SCC47, UM-SCC104 and UD-SCC2	*In-vitro* study	Oral cavity, hypopharynx and tongue	Cross-talk between HER3 and HPV oncoproteins E6 and E7 maintains AKT signaling	Reference
Salazar -Ruales et al. (2018)	Patient samples (*n* = 108)	Case control study	All sites of H&N region	Upregulation of miR-205-5p, miR-122-5p, miR-124-3p, and miR-146a-5p	Downregulation of miR-205-5p, miR-122-5p, miR-124-3p, and miR-146a-5p
	Controls (*n* = 108)				
	HPV positivity (12.7%)				
Chen et al. (2018)	TCGA (*n* = 516)	Cohort study	Oropharynx, larynx	Genes associated with immune-associated processes were upregulated	Reference
	HPV positivity (8.7%)			Greater numbers of infiltrating B and T cells and fewer neutrophils	
	Independent HNSCC datasets (*n* = 78)			Predominated cytotoxic T cell subtypes	
	HPV positivity (30.8%)			Higher ratio of M1/M2 macrophages	
Xiao et al. (2018)	Patient samples (*n* = 94)	Prospective study	Oral cavity, oropharynx, larynx	HPV-related tumors exhibited lower fatigue and inflammation	HPV-unrelated tumors experienced persistently high levels of fatigue and inflammation
	HPV positivity (53.19%)				
Beaty et al. (2019)	Patient samples (*n* = 77)	Clinical trial prospective cohort study	Oropharynx	PIK3CA mutation was significantly associated with disease recurrence and worse DFS.	Reference
Fleming et al. (2019)	Patient samples (*n* = 35)	Cohort study meta analysis	All sites of H&N region	Reference	Elevated expression of genes associated with glycolysis and oxidative phosphorylation
	HPV positivity (31.4%)				

Abbreviations: ASNase- Acid sphingomyelinase activity, CYLD-Cylindromatosis Lysine 63 deubiquitinase, DFS-Disease Free Survival, DMRs-Differentially Methylated Regions, FFPE- Formalin Fixed Paraffin Embedded, HNSCC-Head And Neck Squamous Cell Carcinoma, OCC-Oral Cavity Carcinoma, OPC-Oropharyngeal Carcinoma, OPSCC- Oropharyngeal Squamous Cell Carcinoma, PBLs-Peripheral Blood Lymphocytes, TRAF3-Tumornecrosis Factor Receptor-Associated Factor 3, UADT- Upper Aero Digestive Tract.

In the proliferative cell signaling pathway, HPV-positive HNC showed elevated expression of HER2, HER3, and HER2:HER3, and HER3:PI3K complex. In contrast, HPV-negative HNC showed higher expression of EGFR (HER1), which is responsible for resistance to EGFR inhibitors (Pollock et al., 2015). HPV-positive HNC was PI3K inhibitor resistant due to abundance of E6 and E7 oncoproteins. A crosstalk among PI3K, HER3, and E6/E7 oncogenes was reported (Brand et al., 2018). Differential regulation of several microRNAs was observed in HNC, miR-205-5p, miR-122-5p, miR-124-3p, and miR-146a-5p that were upregulated in HPV-positive HNC. In contrast, these miRNAs were downregulated in HPV-negative HNC (Salazar-Ruales et al., 2018).

Based on transcription milieu, HNC showed constitutively active nuclear factor-κB (NF-κB) irrespective of their HPV status. However, a detailed molecular dissection of the constitutively active NF-κB complex showed the presence of p50:p65 heterodimer in HPV-positive tumors, whereas homodimer of p50:p50 was found in HPV-negative tumors (Mishra et al., 2006; Gupta et al., 2018). Similarly, in HNC tumors for constitutively active AP-1, JunB and JunD were involved with c-Fos and Fra-2 in HPV-positive HNC, whereas in HPV-negative HNC, c-Jun was the major binding partner (Gupta et al., 2015).

STAT3, another transcription factor that is linked with carcinogenic outcome, was strongly associated with HPV-negative HNC and was characteristically low in HPV-positive tumors (Gaykalova et al., 2015; Verma et al., 2017). SOX2 amplification was observed in HPV-negative HNC, while there was no amplification in HPV-positive HNC (Schrock et al., 2014). HPV-positive HNCs were immunologically more active with high infiltration of T and B lymphocytes and myeloid dendritic cells, and had higher M1-type macrophages along with high chemokine production and PD1 expression (Partlova et al., 2015; Chen et al., 2018). A detailed discussion of various differentially expressed carcinogenically relevent genes in HPV-positive and HPV-negative HNC that contribute to better prognosis was described earlier (Aggarwal et al., 2020).

### Current Treatment Strategies Against Head and Neck Cancer

Treatment of HNC requires a multi-modality approach depending on the stage and site of the tumor (Marur and Forastiere, 2008). Early tumors are treated with surgery or radiation, whereas intermediate- and late-stage tumors benefit from a combined modality approach. Due to essential requirement of clear margins in surgery, it is an option only for early tumors; still it carries a risk of cosmetic deformity and impaired function (Kofler et al., 2014). A study on the quality of life after oropharyngeal surgery reports high incidence of fatigue, reduced sexuality, difficulty in swallowing and other teeth, salivary gland, and mouth-opening–related problems (Bozec et al., 2018). Surgery also requires additional treatment to reduce the risk of locoregional and distant failure in advanced-stage HNC (Porceddu et al., 2004). Platinum-based chemotherapy has been central in treating HNC. Combinatorial therapies with or without platinum drugs have been proven superior in terms of the response rate and the ability to tackle drug resistance than platinum-based chemotherapy treatment. Targeted therapies using monoclonal antibodies such as cetuximab, against epidermal growth factor receptor (EGFR) either in combination with a standard chemotherapy regimen or as a single agent, have also proven effective to some extent to treat HNC. But these approaches also bear side effects apart from the development of chemoresistance in a short period of time (Price and Cohen, 2012). These therapies have a myriad of debilitating toxic effects such as nephrotoxicity, hepatotoxicity, and cardiotoxicity. Also, various cardiac events have been reported, like arrhythmias, myocarditis, and cardiomyopathy, to congestive heart failure (Hartmann and Lipp, 2003).

Radiation therapy (RT) is often performed as an adjunct to surgery or in concurrence with chemotherapy (Marur and Forastiere, 2016). Wendt et al. reported a 3-year overall survival rate of 24% in RT arm vs. 48% in RT plus CT arm in stage III/IV HNC, whereas the 3-year locoregional control rate was 17% in RT arm and 36% in RT plus CT arm (Wendt et al., 1998). However, a long-term toxicity risk to the salivary glands, pharyngeal constrictor muscles, and thyroid gland, leading to xerostomia, dysphagia, percutaneous endoscopic gastrostomy tube dependence, chronic aspiration, and hypothyroidism, had been observed (Langendijk et al., 2008).

Despite a clear prognostic advantage and better response to therapy, therapeutic management for HPV-positive HNC is almost the same as that of any HPV-negative HNC. Considering the younger age of the patients, there have been efforts to reduce the long-term toxicity of anticancer treatment without risking the survival benefits (Kofler et al., 2014). Reduction in dose of radiotherapy, use of cetuximab (Marur et al., 2017) instead of cisplatin for chemoradiation, and transoral robotic surgery (TORS) are a few efforts to mention that are specifically directed to HPV-positive HNC. Considering HPV-positive tumors to be immunologically active, in recent past, attempts have been made to design PD1-PDL1 immunotherapeutic strategies (Qiao et al., 2020). New cancer immune-prevention treatments include FDA-approved inhibitory antibodies such as pembrolizumab (anti-PD1 mAb), nivolumab (anti-PD1), and ipilimumab (anti–CTLA-4 mAb) (Bauman and Ferris, 2014; Ferris et al., 2018; Mehra et al., 2018; Havel et al., 2019); co-stimulation and co-inhibition pathways (Kuss et al., 2003; Tsukishiro et al., 2003; Baruah et al., 2012; Pardoll, 2012); and check-point blockade therapy (Davis et al., 2016; Muzaffar et al., 2021). A systematic assessment of the cost effectiveness of ICIs showed nivolumab was not cost-effective over chemotherapy for HNC (Verma et al., 2018). Moreover, none of these approaches target HPV. A study attempted to develop Trojan vaccine against HPV could not show significant benefit of therapeutic vaccines against HPV in HNC (Voskens et al., 2012). A recent study showed a chimeric HPV16 E7 DNA vaccine induced prophylactic and therapeutic efficacy in a cervical cancer mouse model, but its effect on HPV-positive HNC remains to be examined (Garza-Morales et al., 2019).

### Therapeutic Challenges in Head and Neck Cancer Management

Despite aggressive treatment and organ preservation with current clinically administered curative therapies, the overall 5-year survival is less than 50% (Forastiere et al., 2013). With existing heterogeneity in the origin, poor response rates and substantial systemic toxicity associated with current standard-of-care treatment of advanced HNC remain a significant challenge (De Lartigue, 2015). As molecular targeted therapies come into clinical use, the great interindividual variability in the efficacy of these compounds highlights the absolute need to determine predictive factors of tumor and toxic responses to these new therapeutic agents (Bozec et al., 2009). Further, patients with locally advanced or recurrent HNC present a separate therapeutic challenge. Treatment options are limited, and morbidity can be substantial. Surgical intervention has debilitating effect on normal daily routine and patient psychology. Recurrent HNC is difficult to treat for multiple reasons, including the effects of prior treatment on tumor cells and normal tissues, as well as the infiltrative and multifocal nature that typically characterizes recurrent disease in this area (Ho et al., 2014).

Limitations of these therapies have prompted clinical and translational research for better chemotherapeutics with less treatment-associated toxicities. Many studies are focusing on biologically active compounds from herbal origin to develop chemotherapeutic agents with fewer side effects and higher efficacy (Seo et al., 2015; Kunnumakkara et al., 2017). Many of these phytochemicals can serve as alternatives for chemotherapy sensitizers (Bharti and Aggarwal, 2017; Huang and Yu, 2017).

### Emerging Chemotherapeutic Phytochemicals/Herbal Derivatives Against Head and Neck Cancer

Phytochemicals have found relevance in HNC therapy because natural compounds provide a cost-effective, safe, and less toxic alternative to synthetic drugs currently in wide use. Effectiveness of various phytochemicals as therapeutic agents has been well documented in the literature, and they are now widely being studied as potential agents to treat and prevent HNC. Many preclinical studies have successfully demonstrated the anticancer activity of pure and well-characterized phytochemicals and herbal derivatives on cells obtained from different HN regions using *in vitro* and *in vivo* experimental systems (Table 4). However, a majority of these studies employed cell lines derived from the oral cavity, so the data may be slightly skewed.

**TABLE 4:** Preclinical studies showing therapeutic phytochemicals/herbal derivatives against tobacco/alcohol-associated HNC.

Bioactive compound/ Herbal derivative	Cell type/Model	Test and oosage	Anti-tumour outcome	Molecular outcome	References
PubChem CID (Class)					
Source					
Curcumin	*In vitro*: **MDA 1986** (OSCC), **Tu 686** (LSCC), **Tu 167**, **JMAR C42** (Floor of mouth OSCC), **MDA 686LN** (OPSCC)	**Cell proliferation:** 10 μM, 50 μM	• Proliferation↓	• NF-κB activation↓	Aggarwal et al. (2004)
CID- 969516			• Apoptosis↑	• Bcl-2↓, cyclin D1↓, IL-6↓, COX-2↓, MMP-9↓	
(Phenolics)			• Arrests cell cycle in G1/S phase		
*Curcuma Longa*					
Gossypol	*In vitro:* **UMSCC-1**, **UMSCC-17B** (Floor of mouth OSCC), Human oral keratinocytes and Normal keratinocytes	**Cell proliferation:**	• Proliferation↓	NA	Wolter et al. (2006)
CID- 3503		UMSCC-1, UMSCC-17B, Human oral keratinocytes and Normal keratinocytes IC50- 3, 6,2, 12.5 μM respectively	• Growth of tumour↓		
Phenolic			• Mitotic rate↓		
*Gossypium arboreum L.*	*In vivo*: NCr-nu/nu mice		• Apoptosis↑		
Berberine	*In vitro:* **HSC-3** (Tongue OSCC)	**Cell viability:**	• Cell viability↓	• Bcl-2↓, BAX↑, p53↑	Lin et al. (2007)
CID: 2353		10 μM	• G0/G1-phase arrest	• Cyt C release	
(Alkaloid)			• ROS↑,Ca^2+^↑, MMP↓, Apoptosis↑		
*Rhizoma coptidis*					
	*In-vitro* study*:* **FADU** (Hypopharyngeal SCC)	**Cell cytotoxicity:**	• Cytotoxicity ↑	• FasL ↑, TRAIL ↑	Seo et al. (2015)
				• Cleaved caspase-8 ↑, cleaved	
		12 or 25 μM for 24 h	• Apoptosis ↑	• caspase-7 ↑	
			• Cell viability ↓	• Bcl-2 ↓, Bcl-xL ↓, Bax ↑, Bad ↑, Apaf-1↑, cleaved caspase-9 ↑, cleaved caspase-3 ↑, PARP ↑	
			• Cell migration ↓		
				• MMP-2 ↓, MMP-9 ↓	
				• ERK, JNK and p38 phosphorylation ↓	
*Physalis angulate*	*In-vitro* study*:* **HSC-3** (OSCC)	**Cell viability:**	• Mitochondrial reductase activity ↓	• ROS ↑	Lee et al. (2009)
			• Apoptosis ↑	• Bcl2 ↓, Bax ↓, AIF ↓, cytochrome c ↓, proform caspase-3 protein levels ↓, caspase-9 ↑, proform caspace-4 protein levels ↑, MMP attenuated	
(Crude extract)		IC50: 10 μg/ml	• Oxidative stress ↑		
			• Loss of cell function ↑		
				• ORP150 ↓, HSP70 ↑	
			• S- and G2/M-phase arrest	• HO-1 ↑, SOD ↑	
			• Mitochondrial function impaired		
Guggulsterone	*In vitro:* **SCC-4** (Tongue OSCC), **HSC-2** (OSCC)	**Protein expression:**	NA	• Phosphorylation of p65, IkBα & STAT3, NF-κB↓	
CID- 6450278		50 μM			
(3-hydroxy steroid)				• IL-6↓ , COX-2↓	
*Commiphora mukul*				• Expression VEGF↓	
Proanthocyanidins	*In vitro:* **UMSCC-1**, **UMSCC-5** (Floor of mouth OSCC), **FaDu** (Hypopharyngeal OSCC), **OSC-19** (Tongue OSCC), **Beas-2B** (Bronchial Epithelium transformed with Ad12-SV40 2B)	*In vitro:* IC50- 101 nM, 67 nM for	• Cell viability↓	• Cyclin D1↓ and Cyclin D2↓	Prasad and Katiyar, (2012)
CID- 108065		24 and 48 hrs, respectively	• Apoptosis↑	• cdk2↓, cdk4↓, cdk6↓	
			• G1 phase arrest	• Cip1/p21↑ and Kip1/p27↑	
		*In vivo:* 0.5%, w/w	• 61% less tumor volume (p<0.001)	• Bax↑, Bcl-2↓, caspase 3↑ and poly(ADP-ribose)polymerase↑.	
(Phenolic)					
*Vitis vinifera*					
	*In vivo:* athymic nude mice				
Wogonin	*In vitro:* **NPC-TW076, NPCTW039** (NPSCC)	**Autophagy:** 50 μM	• Autophagy↑	• P13K/Akt↓, mTOR/P70S6K↓, c-Raf/ERK↓	Chow et al. (2012)
CID- 5281703			• Apoptosis↑		
(Phenolic)					
*Scutellaria baicalensis*					
Caffeic Acid Phenethyl Ester	*In vitro:* **TW2.6** (OSCC)	**Cell proliferation:** IC50- 83.8, 46.6, and 18.8 μM for 24, 48, and 96 h treatment, respectively	• Cell proliferation↓, colony formation↓	• Akt↓, GSK3β↓, FOXO1↓, FOXO3a↓, NF-κB↓, Rb↓, Skp2↓, cyclin D1↓, p27Kip↑	Kuo et al. (2013)
CID- 5281703			• Apoptosis↑		
(Phenolic)			• G1 phase↓, G2/M phase↑ cell population		
*Populus nigra L*.					
Ellagic acid	*In vitro:* **HSC-2** (OSCC), **HF-1** (Normal fibroblasts)	**Cytotoxicity:** (IC50- 260 & 142 μM on 2^nd^ and 3^rd^ day of exposure, respectively)	• Apoptosis↑	• Caspase 3/7 ↑	Weisburg et al. (2013)
CID- 5281855				• Cleavage of poly ADP ribose polymerase	
(Phenolic)					
*Rubus occidentalis*					
Cucurbitacin	*In vitro:*	**Cytotoxicity:** IC50- 3.7 μM	• Sub G0/G1 phase arrest	• Caspase 3 Activation	Hung et al. (2013)
CID- 5281316	**SAS** (Tongue OSCC)		• Apoptosis↑	• MMP↓	
(Terpene)					
*Cucumic melo L*					
BME	*In vitro:* **Cal-27** (Tongue OSCC), **JHU-22**, **JHU-29** (Laryngeal SCC)	*In vitro*: 1% BME	• Cell proliferation↓	• c-Met signaling pathway↓	Rajamoorthi et al. (2013)
(crude extract)		*In vivo:* 100 μl BME (0.1 g/ml) for 5 days	• Tumor growth and volume↓	• Mcl-1, pSTAT3, cMyc expression↓	
*Momordica charantia*			• Keratinocyte formation↓, mitosis↓	• cyclinD1↓, survivin↓, cell arrest	
	*In vivo:* BALB/c athymic nude mice			• MCM2↓	
Honokiol	*In vitro:* **SCC-1, SCC- 5** (Floor of mouth OSCC), **OSC-19** (Tongue OSCC), **FaDu** (Hypopharyngeal SCC)	*In vitro*: ∼60 μM for 24, 48 and 72 hrs	• Cell viability↓	• Expression levels of Cyclins: D1, D2, and Cdks: 4 and 6↓	Singh et al. (2015)
CID-72303			• Apoptosis↑		
(Phenolic)		*In vivo:* 100 mg/kg body weight (mw-266.3)		• EGFR↓, mTOR and their downstream signalling molecules↓	
*Magnolia officinalis*					
Cepharanthine	*In vitro:* **CNE-1, CNE-2** (NPSCC)	**Cell proliferation:**	• Cell proliferation↓	• DNA repair genes ↓	Liu et al. (2015)
CID- 10206		IC50 for CNE-1 and CNE-2: 20 and 32 nM after 48 hrs, respectively	• G1 phase arrest		
(Alkaloid)					
*Stephania cepharantha*					
MEAG	*In vitro:* **MC3** (Chronic myelogenous leukemia), **HN22** (OSCC)	**Cytotoxic effect:**	• Mitochondria mediated apoptosis↑	• MMP↓, Cyt C release, caspase 9 ↑, t-Bid↑, cleaved caspase-8↑, DR5↑.	Lee et al. (2016)
(Crude extract)		IC50 for MC3 and HN22: 6.5 and 4.6 μg/ml, respectively			
(Terpenes)			• Nuclear condensation and fragmentation		
*Withania somnifera*					
Goniothalamin	*In vitro:* **H400** (OSCC)	**Cytotoxic effect:**	• Cell viability↓	• MMP↓, Cyt C release	Li et al. (2016)
CID- 6440856		IC50: 8.9 nM after 72 h	• Cell proliferation↓	• NF-κB activation↓	
(Phenolic)			• Apoptotic-like morphology (cell shrinkage, dense cytoplasm, blebbing of cell surface)		
*Goniothalamus marcrophyllus*					
			• S phase arrest.		
Lupeol	*In vitro:*	**Cell viability:**	• G1 phase arrest	• Expression p53↑, Bax↑, CDKN2A↑, CyclinD1↓, Ki67↓	Bhattacharyya et al. (2017)
CID- 259846	**HEp-2** (Human papillomavirus-related endocervical adenocarcinoma), **UPCI:SCC131** (Floor OSCC)	IC50 for Hep-2 and SCC131: 53.5 and 52.4 μM after 24 hrs, respectively	• Apoptosis↑ via intrinsic pathway		
(Terpene)			• Cell viability↓	• Caspase 3 activation	
*Camellia japonica*					
	*Ex vivo:* Fresh HNSCC tumor tissues				
Icaritin	*In vitro:* **KB** (Human papillomavirus-related endocervical adenocarcinoma), **SCC9** (Tongue OSCC)	**Cell viability:**	• Mitochondria mediated apoptosis↑	• MMP↓, Cyt c release	Jin et al. (2017)
CID- 5318980		∼20 μM for 24 and 48 h		• miR-124↑	
(Phenolic)				• Sp1/DNMT1 signaling↓	
*Epimedium grandiflorum*					
*Osmuda regalis* root	*In vitro:* **FaDu** (Hypopharyngeal SCC), **HLaC78, HLaC79, HLaC79-tax** (LSCC)	**Cytotoxicity:**	• Cell growth↓	• CLEC3B↓, KAL1↓, MMP 11↓, MMP 15↓, MMP2↓	Schmidt et al. (2017)
(crude extract)		IC50 for HLaC79, FaDu, HLaC79-Tax and HLaC79: 21.4, 8.5, 20.6 and 9.9 μg/ml, respectively	• Apoptosis↑		
*Osmuda regalis*			• Invasion↓	• Integrin such as ITGB3↑, ITGA1↑, ITGAM↑	
				• metastasis genes such as CTFG↑, PPIA↑, SELP↑, VCAN↑	
Piperine	*In vitro:* **KB** (Human papillomavirus-related endocervical adenocarcinoma)	**Cell proliferation:**	• Apoptosis↑	• ROS↑, MMP↓	Siddiqui et al. (2017)
CID- 638024		IC50: 124 μM for 24 h	• Cell viability↓	• Caspase 3 activation	
(Alkaloid)			• Cell growth↓		
*Piper nigrum*			• Cellular morphological changes		
			• G2/M phase arrest		
Embelin	*In vitro:* **SCC25**, **Cas9-22**, **YD10B** (Tongue squamous cell carcinoma)	**Cytotoxicity:**	• Cell viability↓	• MMP↓, caspase 9 & 3↓	Lee et al. (2017)
		0–300 μM for 24 h; cell specific effect	• Condensed fragmented nuclei	• p62/SQSTM1↓	
			• Autophagic vacuoles appears	• Conversion↑ LC3-I to LC3-II	
CID- 32798					
(Phenolic)					
*Embelia ribes*					
Dihydroartemisinin	*In vitro:* **Cal-27** (Tongue OSCC)	**Cell proliferation:**	• Autophagosomes↑	• LC3-II ↑, Bevlin-1↑, γH2AX foci↑	Shi et al. (2017)
CID- 3000518		IC50: 24.4 μM for 24 h	• Oxidative stress↑	• STAT3 activation↓ and disrupted p-STAT3 nuclear translocation	
			• DNA double-strand break		
(Sesquiterpene lactone)			• Weight and volume of Xenograft tumor↓ by 56.58%		
*Artemisia annua*					
β-Elemene	*In vitro:* **YD-38** (Gingival OSCC)	**Colony formation and Apoptosis:**	• Cell proliferation↓	• Expressions of STAT3↓, p-STAT3↓, p-JAK2↓, and Bcl-2↓, Bax↑ and caspase-3↑	Huang and Yu, (2017)
CID- 6918391	*In vivo*: BALB/c nude mice		• Apoptosis↑		
(Terpene)		195 nM β-Elemene			
*Curcuma wenyujin*				• Block JAK2-STAT3 pathway	
Thymol	*In vitro:* **Cal27, SCC-4, SCC-9** (Tongue OSCC)	**Cytotoxicity:**	• Cell viability↓	• c-PARP↑, MMP dysfunction	De La Chapa et al. (2018)
CID- 6989	*In vivo:* Athymic nu/nu mice	2.3 µM	• No colony formation		
(Terpene)			• *In vivo* tumor growth↓		
*Thymus vulgaris*			• Apoptosis↑		
Tanshinone	*In vitro:* **SCC-9** (Tongue OSCC)	**Cell viability:**	• Apoptosis↑	• LC3-I↑	Qiu et al. (2018)
CID- 114917	*In vivo:* BALB/c-nu	IC50: 17.5 μM.	• Autophagy↑	• Beclin-1/Atg7/Atg12-Atg5 pathway↑	
(Abietane diterpenoid)			• Growth of solid tumors in vivo	• PI3K/Akt/mTOR pathway↓	
*Salvia miltiorrhiza*					
Oridonin	*In vitro:* **UM1, SCC25** (Tongue OSCC)	**Cell proliferation:**	• G2/M phase arrest	• Bax/Bcl-2↑	Yang et al. (2018b)
CID- 5321010		IC50 for SCC25, UM1, UM2, HSC3 and Ca127: 9.1, 8.2, 10.6, 15.4 and 9.6 μM, respectively.	• Apoptosis↑	• Cyclin B1↓, pCDK1↑, cyclin D1↑, cyclin D3↑, p21↑ and cyclin A2↑	
(Terpene)					
*Rabdosia rubescens*				• Activates caspase-3, caspase-9 and PARP-1	
				• P13K/Akt/mTOR pathway↓	
Epigallocatechin-3-gallate	*In vitro:* **HSC** (Tongue OSCC)	**Cell proliferation:**	• Cell viability↓	• Caspase‑3 and -7↑	Yoshimura et al. (2019)
	*In vivo:* BALB/c nude (nu/nu) mice	IC50 value at 24, 48 and 72 h were >100, 43.2 and 39.3 μM, respectively	• G1 phase arrest	• miR-22↑	
CID- 65064			• Apoptosis↑		
(Phenolic)			• Tumor size↓ 45.2%		
*Camellia sinensis*					
Quercetin	*In vitro:* **hNOK** (Human normal oral keratinocytes), **Tc8113, SAS** (Tongue OSCC)	**Cell viability:**	• Cell viability↓	• miR-22/WNT1/Beta-catenin pathway↓	Zhang et al. (2019)
(Phenolic)		CC_50_ for hNOK, Tc8113 & SAS: 298.6, 48.7 & 44.3 mM, respectively	• Tumor volume and weight↓		
CID: 5280343					
*Allium cepa L.*					
	*In vivo*: BALB/c nu/nu mice				
Ursolic Acid	*In vitro:* **Ca9-22** (Tongue OSCC), **SCC2095** (OSCC)	**Cell proliferation:**	• Caspase-dependent apoptosis	• Akt/mTOR/NF-κB signaling↓, ERK↓, and p38↓	Lin et al. (2019)
CID- 64945		IC50 for UA, Ca9-22: 11.5 and 13.8 μM, respectively	• Autophagy↑, autophagosomes↑		
(Terpene)			• Migration↓, Invasion↓	• LC3B-II conversion, p62↑	
*Salvia rosmarinus*				• Proteolytic activity of MMP-2↓	
Shikonin	*In vitro:* **5-8F** (NPSCC)	**Cell proliferation:**	• Necroptosis↑	• Necrostatin-1↑	Liu et al. (2019)
CID- 479503	*In vivo:* BALB/c nude mice	IC50: 7.5 μM after 6 h	• *In vivo* tumor growth↓	• RIPK1↑, RIPK3↑, MLKL↑	
(Hydroxy-1,4-naphthoquinone)				• Caspase-8 and -3↑	
				• ROS↑	
*Lithospermum erythrorhizon*					
Chrysophanol	*In vitro:* **FaDu** (Hypopharyngeal SCC), **SAS** (Tongue OSCC)	**Cell viability:**	• Cell viability↓	• ROS↑	Hsu et al. (2020)
CID- 10208		IC50 for FaDu and SAS: 9.6 ± 1.3 and 12.6 ± 2.1 μm at 24 h, respectively	• G1 phase arrest	• Expression of procaspase 3↓, cyclin D1↓, CDK4↓, CDK2↓, cdc2↓	
(Phenolic)			• Metastasis↓, EMT↓		
*Rheum rhabarbarum*					
Moscatilin	*In vitro:* **FaDu** (Hypopharyngeal SCC)	**Cell cytotoxicity:**	• Cell viability↓	• Activation of caspases-3,-8,-9,-7↑	Lee et al. (2020)
CID- 176096		IC50: 1.4 μM at 72 h	• Cell proliferation↓	• MMP↓, Cyt C release	
(Phenolic)			• Apoptosis via intrinsic as well as extrinsic pathway	• JNK pathway↓	
*Dendrobium sp.*					
Demethoxycurcumin	*In vitro:*	**Cell proliferation:**	• Cell viability↓	• cIAP1/XIAP↓, heme oxygenase-1↑	Chien et al. (2020)
	**SCC-9, HSC-3** (Tongue SCC)	IC50: 50 μM	• Cell proliferation↓	• Caspase-3↑, -9↑, -8↑, p38-MAPK-HO-1 signaling↑, MAPK↑, JNK1/2↑	
CID- 5469424			• G2/M phase arrest		
(Phenolic)			• Morphological changes		
*Curcuma Longa*					

Abbreviations: Akt- Protein kinase B; Apaf-1- Apoptotic protease activating factor 1; Atg7- Autophagy related 7; Bad- BCL2-associated Agonist of cell Death; Bcl-2- B-cell lymphoma 2; BME- Bitter Melon Extract; Cdc2- Cell division control 2; Cdk2- Cyclin-dependent kinase 2; CDKN2A- Cyclin-dependent kinase inhibitor 2A; cIAP- Calf Intestinal Alkaline phosphatase; Cip1- CDK interacting protein 1; CLEC3B- C- type lectin domain family three member B; c-Met- tyrosine-protein kinase Met; COX-2- Cyclooxygenase-2; c-PARP- Cleaved Poly (ADP-ribose) polymerase; c-Raf-c- Rapidly Accelerated Fibrosarcoma; CTGF- Connective tissue growth factor; Cyt C- Cytochrome complex; DNMT1- DNA (cytosine-5)-methyltransferase 1; DR5- Death receptor five; ERK- Extracellular-signal-regulated kinase; FasL- Fast ligand or cell death receptor; FOXO1- Forkhead box protein O1; GSK3β- Glycogen synthase kinase three beta; H&N- Head and neck; HO-1- Heme oxygenase 1; HSP70–70 kilodalton heat shock proteins; IkBα- I-kappa-B-alpha; ITGA1- Integrin alpha-1; ITGAM- Integrin alpha M; ITGB3- Integrin beta three; JNK- c-Jun N-terminal kinase; Kip1- kinase inhibitor 1; LC3- Microtubule-associated protein 1A/1B-light chain three; MAPK- Mitogen-activated protein kinase; Mcl-1- Myeloid leukemia cell differentiation protein 1; MCM2- Minichromosome maintenance protein complex 2; MLKL- Mixed lineage kinase domain-like pseudokinase; MMP- Matrix metallopeptidase; mTOR-mammalian target of rapamycin; NF-κB- Nuclear factor kappa light chain enhancer of activated B cells; ORP150–150-kDa oxygen-regulated protein; P13K- Phosphatidylinositol 3-kinase; P70S6K- 70-kDa ribosomal protein S6 kinase; PARP- Poly-ADP ribose polymerase; JAK- Janus kinase; PPIA- Peptidylprolyl isomerase A; Rb- Retinoblastoma protein; RIPK1- Receptor-interacting serine/threonine-protein kinase 1; ROS- Reactive oxygen species; SOD- Superoxide dismutases; Sp1- Specificity protein 1; SQSTM1- Sequestosome-1; STAT3- Signal transducer and activator of transcription three; t-Bid-truncated BH3 interacting-domain death agonist; TRAIL- TNF-related apoptosis-inducing ligand; VEGF- Vascular endothelial growth factor; WNT1- Wingless-related integration site 1; XIAP- X-linked inhibitor of apoptosis protein; yH2AX- Phosphorylated X-linked inhibitor of apoptosis protein.

**FIGURE 3:**
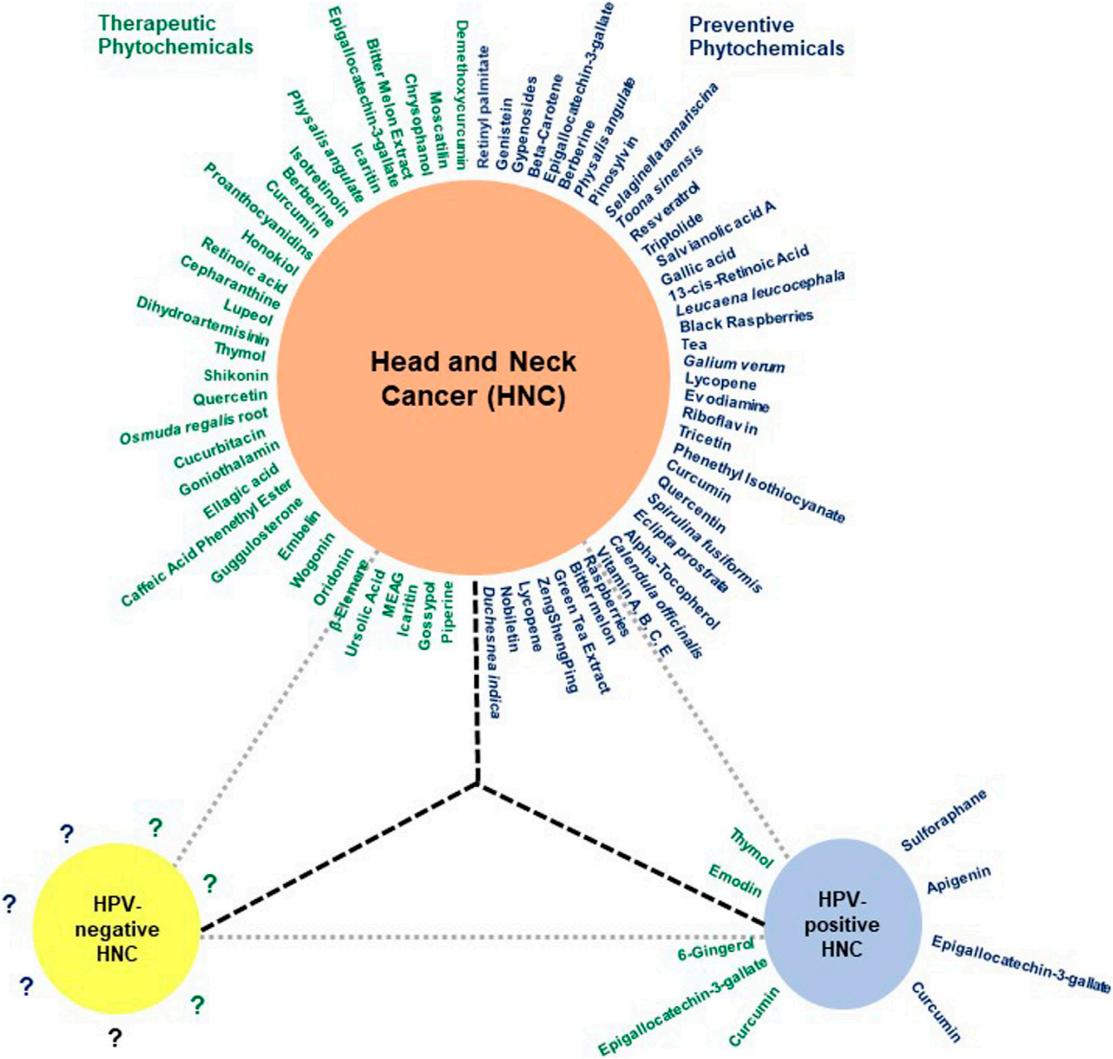
The huddle of chemopreventive and chemotherapeutic phytochemicals in HNC. Schematic diagram showing phytochemicals with chemopreventive and chemotherapeutic properties in **blue** and **green,** respectively. Since most reports addressing the effect of herbal derivatives on HNC lacked HPV-related information, the data may not be directly applicable to HPV-negative HNC and require prior validation in HPV-negative HNC cells. Additionally, the HPV-positive HNC remains a poorly explored area.

A range of phytochemicals showed anticancer activity against different HNC cells over 2 decades (Figure 3). Phytochemicals like thymol, oridonin, shikonin, and moscatilin with potent dose-dependent antiproliferative activity showed IC50 values lower than 10 μM over a wide range of HNC cell types. A detailed investigation of molecular mechanisms revealed targeting of key cellular carcinogenic pathways, namely, MAPK/JNK/p38 (role of ROS), NF-κB, EGFR/JAK2/STAT3, P13K/Akt, mTOR/P70S6K, c-Raf/ERK, GSK3β, FOXO1, FOXO3a, and p53, that concurrently operate in HNC and contribute to cancer progression and treatment resistance.

NF-κB is a family of transcription factors (TFs) comprising c-Rel, RelA (p65), RelB, NF-κB1 (p50 and p105), and NF-κB2 (p52), which plays important roles in immunity, inflammation, cell proliferation, survival, and differentiation (Oeckinghaus and Ghosh, 2009). Many basic and clinical studies demonstrated aberrantly expressed and constitutively expressed NF-κB in HNC with its contribution to cancer cell survival and proliferation, and poor survival of patients (Mishra et al., 2006; Monisha et al., 2017; Verma et al., 2017). Cigarette smoke phosphorylates IκBα, which in turn activates NF-κB (Anto et al., 2002). Early evidence of phytochemicals like curcumin showing anticancer action on HNC came from abrogated IκBα kinase (IKK) which inhibited NF-κB activation and cell survival/proliferation genes such as cyclin D1, Bcl-2, IL-6, COX-2, and MMP-9 (Aggarwal et al., 2004). Based on a similar approach, blocking activity of NF-κB, or its downstream molecules, therapies were designed to downregulate cell growth and metastasis. Caffeic acid phenethyl ester (CAPE) and goniothalamin inhibited NF-κB-p65 activity in a potential primary and metastatic OSCC (Kuo et al., 2013; Li et al., 2016).

STAT3, a central transcription factor and known oncogene, works downstream of EGFR, and TGFα signaling also plays a key carcinogenic role in HNC (Song and Grandis, 2000). Guggulsterone, a biosafe nutraceutical, phosphorylated p65 and inhibited tobacco smoke and nicotine-induced NF-κB and pSTAT3 proteins and their downstream targets COX-2 and VEGF (Macha et al., 2011). Dihydroartemisinin is a known phytochemical, which is effective as an antimalarial agent, induces DNA double-strand break and promoted oxidative stress, and decreases pSTAT3 nuclear localization which successively increases autophagic cell death (Shi et al., 2017).

In 90% HNC, the PI3K/AKT/mTOR pathway is upregulated (Marquard and Jucker, 2020). Whenever ligand-like growth factors bind with RTKs, they dimerize and lead to the activation of intercellular tyrosine kinase. PI3K partially activates Akt through PIP3 and PIP2. Then to stimulate full activity of Akt, mTORC2 phosphorylates its carboxy-terminal. Akt functions by phosphorylation that leads to the activation or suppression of many proteins involved in cell proliferation, growth, and cell motility (Brazil and Hemmings, 2001; Chaisuparat et al., 2016). Wogonin, a flavonoid compound, has anticancer activity which induces autophagy by LC3 I/II cleavage and inhibits mTOR/P70S6K and Raf/ERK, which in turn inactivates PI3K/Akt and induces apoptosis in NPC cells (Chow et al., 2012). Urosolic acid downregulated Akt/mTOR signaling and expression of NF-κB, which further downregulates ERK and MMP-2 in OSCC cells (Lin et al., 2019).

Loss of carcinogenic signaling was associated with reduced cell survival mechanisms. Honokiol, a phytochemical from *Magnolia* plant, reduced the level of Bcl-xL protein, while Bax expression in xenograft HNC tumors increased. It also reduces the expression of mTOR and its downstream p70S6K (Singh et al., 2015). Similarly, (-)-gossypol, a polyphenol, was reported to bind to Bcl-xL that inhibited HNC proliferation (Wolter et al., 2006).

Antiproliferative activity of phytochemicals was associated with various degrees of cell cycle arrest in most of these studies. Cell cycle–regulating molecules such as cyclins and cdks were downregulated by oridonin, chrysophanol, lupeol, honokiol, and proanthocyadins. Piperine, a nitrogenous pungent substance, induced cell cycle arrest in the G2/M phase and induced apoptosis by changing mitochondrial membrane potential and by activating caspase-3 (Siddiqui et al., 2017). Chrysophanol, a secondary metabolite, downregulated the expression of cyclinD1, CDK4, cdc2, and CDK2, and arrested cell cycle at the G1 phase. It also induced cell death by ROS production (Hsu et al., 2020). Similarly, lupeol induced cell cycle arrest in the G1 phase by increasing the expression of p53, Bax, and CDKN2A, and downregulating cyclin D1 (Bhattacharyya et al., 2017). Oridonin, a bioactive diterpenoid, induced apoptosis by regulating Bax/Bcl-2 and activating caspases. It also decreased cell proliferation by downregulating PI3K/Akt/mTOR pathways. By regulating cyclins, it arrested cells in the G2/M phase (Yang et al., 2018b). Even though the end effect was antiproliferative, the mechanism of action of these phytochemicals differed significantly.

A family of cysteine proteases known as caspases regulates apoptosis. Targeting these caspases can induce apoptosis in OSCC. Demethoxycurcumin, a curcumin analog, induced apoptosis in tongue SCCs by upregulating caspase-3, -9, and -8. It also regulated p38-MAPK-HO1 signaling, MAPK, and JNK1/2 (Chien et al., 2020). Shikonin induced necroptosis in NPC *via* upregulating the expression of RIPK1/RIPK3/MLKL, caspase-3, and -8, and increasing ROS production (Liu et al., 2019). Ellagic acid induced apoptosis by upregulating caspase-3 and -7 (Weisburg et al., 2013). Curcurbitacin, embelin, and proanthacyadins induced apoptosis by attenuating mitochondrial membrane potential and by regulating the activity of Bcl-2, Bcl-xL, and Bax in cells (Prasad and Katiyar, 2012; Hung et al., 2013; Lee et al., 2017).

In *in vivo* studies, the phytochemicals were tested in murine models, where nude mice were implanted with OSCC cell lines. These mice were used to measure the effect of phytochemical on tumor growth. Tumors from euthanized mice were examined for their size and volume. ECGC (Yoshimura et al., 2019), gossypol (Wolter et al., 2006), quercentin (Zhang et al., 2019), proanthocyadins (Prasad and Katiyar, 2012), tanshinomes (Qiu et al., 2018), shikonin (Liu et al., 2019), β elemene (Huang and Yu, 2017), and bitter melon extract (Rajamoorthi et al., 2013) depicted reduction in size and volume of tumor xenografts, and inhibition of xenograft growth. Inhibition of growth was also observed in the *ex vivo* study with lupeol.

Some of the phytochemicals were also tested in clinical trials; however, these studies are very limited (Table 5) and emphasize an urgent unmet need in this area to harness the translational potential of emerging phytochemicals. Lippman et al., 1988 conducted a phase II randomized study with 13-cis-retinoic acid (isotretinoin) (3 mg/kg/day) and methotrexate (15 mg/m^2^ on the first three days in a 3-week cycle) among 40 patients with advanced SCCs. They achieved a response rate of 16% with isotretinoin, which included a complete response, a partial response, and a minor response. In the methotrexate-treated group, however, the response rate was 5%. The median survival rate from the start of treatment was also lower in the methotrexate group (4 months) than that in the isotretinoin group (4.5 months) (Lippman et al., 1988). Another phase I study with isotretinoin by Weisman et al., 1998 reported its strong synergetic relationship with cisplatin. The maximum tolerated dosage as determined by the study (20 mg/day) was able to attain a complete response at the primary site in all of the 10 evaluable patients (Weisman et al., 1998). There are very few clinical trials on therapeutic potential of phytochemicals in HNC because of lack of interest from pharma industry due to low cost of the molecules, and clinical trial requires a lot of investments. Also, HNC patients with advance stage tumor do not participate in therapeutic clinical trials as it may risk the available therapeutic benefits of existing therapies; however, use of phytochemicals as adjunct therapies may prove beneficial in long run as they will not compromise the benefits of participating patients. Nevertheless, more *in vivo* studies are needed to screen promising leads into clinical trials.

**TABLE 5:** Clinical studies in therapeutic phytochemicals/herbal derivatives against HNC.

Phytochemical/Herbal extract	Type of study	Study subject	Dosage, treatment duration (follow up)	Criteria (I: Inclusion/E: Exclusion)	Outcomes	Adverse effects	References
Isotretinoin	Phase II randomized trial	*n* = 40	Isotretinoin: 3 mg/kg/day	**Inclusion**	• Complete response: 1	• Moderate, consisting primarily of mucocutaneous toxicity with no life-threatening problems	Lippman et al. (1988)
		Evaluable: 38	Methotrexate: 15 mg/m^2^ on days 1,2,3 of a 3 weeks cycle	• Measurable histologically confirmed locally advanced or metastatic SCCA of the head and neck	• Partial response: 1		
		Isotretinon:Methotrexate::19:19	For atleast 6 weeks. Evaluation: Every 3 weeks	• Karnofsky performance score of >50%	• Minor response: 1		
		Age- 42–76 years		• Life expectancy of at least 8 weeks	• Total response rate: 16%		
		Gender bias-M:F::33:5		• Adequate renal and liver function (creatinine <2.0 mg/dl and bilirubin <2.0 mg/dl)	• Median survival rate from start of treatment with isotretinoin: 4.5 months		
				• Must not have received			
				• Radiation therapy within six weeks prior to starting this trial			
				**Exclusion**			
				• Women with reproductive capacity			
				• Persons taking large doses of vitamin a (>25,000 IU per day)			
Retinoic acid	Phase I open trial	*n* = 15	Starting dose of 20 mg/day. Increased in increments of 20 mg 20 mg/day, 40 mg/day, 60 mg/day	**Inclusion**	• The maximum tolerated dosage in this setting for CRA was 20 mg/day	• Dose limiting toxicity: Neutropenia only; observed in 1/6 patients treated at 20 mg dose and 3/20 patients treated with 40 mg dose and 1 patient treated at 60 mg dose	Weisman et al. (1998)
		RA20:RA40:RA60::6:8:1	All 7 days prior to chemoradiation therapy with high dose cos-platinum (150 mg/m^2^)	• Older than age 18	• Of 10 patients with fully evaluable data, all achieved a complete response at the primary site and 9 had a complete response in the neck	• Grade III and one had grade IV stomatitis in 6 and 1 patients respectively	
		Age- 40–74 years	Tumor responses were determined every 2 weeks and drug toxicities evaluated weekly	• Untreated, biopsyproven, squamous cell carcinoma of the upper digestive tract	• Pretreatment with retinoic acid results in stronger synergy than concurrent drug exposure alone	• Thrombocytopeniamild dry mouth and mild dry skin	
		No. of patients with stage IV disease: 15		• No evidence of distant metastases			
		No. of patients with neck disease (N3/N4): 12		**Exclusion**			
		Types of tumor- T3:T4::3:12		Patients with another malignancy diagnosed within 5 years of the head and neck malignancy			
		Fully evaluable and completed treatment: 10					

### Emerging Chemopreventive Phytochemicals/Herbal Derivatives Against Head and Neck Cancer

Cancer chemoprevention refers to the use of agents to retard the progression of carcinogenesis, reverse, or inhibit it. The aim of chemoprevention is to lower the risk of developing invasive or clinically significant disease. Chemopreventive phytochemicals thus seek to occasion a chemopreventive response when the primary tumors have not reached a critical size, or seek to block and reverse development of a diagnosed premalignant tumor, or prevent metastasis and growth of metastatic tumors (Tosetti et al., 2002). Angiogenesis, which refers to the biological process of vessel formation, also plays a crucial role in cancer progression. Angiogenesis is also responsible for transition of a dormant tumor to a malignant state (Sogno et al., 2009). An early intervention could possibly prevent cancer formation by regulating “angiogenic switch,” the point at which the tumor induces angiogenesis. Thus, angiogenesis is a critical target for chemoprevention (Tosetti et al., 2002).

A battery of phytochemicals reportedly possess cell invasion, migration, angiogenesis, and metastasis inhibitory activities (Table 6). These phytochemicals exhibit these antitumor activities by regulating the expression of various molecules such as metalloproteinases (MMPs), especially MMP-2 and MMP-9, which affect cancer migration and invasion. Some MMPs also exhibit proangiogenic properties as they can activate proangiogenic factors such as VEGF, and angiopoietin (Folgueras et al., 2004). These phytochemicals were also observed to regulate the MAPK/ERK pathway, which plays a crucial role in cell proliferation (Chen et al., 2019).

**TABLE 6:** Pre-clinical studies in emerging chemopreventive phytochemicals/herbal derivatives against HNC.

Bioactive compound/Herbal derivative	Cell type/Model	Test and dosage	Anti-tumor outcome	Molecular outcome	References
PubChem CID (class) source					
Genistein	*In vitro study:*HSC-3 OSCC)	*In vitro* invasion assay: 27.3 μg/ml for 24 h	• Invasion↓	• VEGF mRNA↓	Myoung et al. (2003)
CID-5280961 (Phenolic)	*In vivo* study: Female BALB/c nude mice	*In vivo* assay: 0.5 mg/kg daily	• Gelatinolytic activity↓		
Epigallocatechin-3-gallate	*In vitro:* OC2 (OSCC)	Invasion and migration assays: >40 μM	• No cytotoxic effect	• MMP-2↓, MMP-9↓, and uPA↓	Ho et al. (2007)
CID-65064 (Phenolic)			• Migration↓		
*Camellia sinensis*					
Berberine	*In vitro:* SCC-4 (tongue OSCC)	Wound-healing assay: 125 μM for 48 h	• Cell migration and invasiveness ↓	• MMP-2↓, MMP-9↓, u-PA↓, FAK↓, p-p38↓, *p*-JNK↓, *p*-ERK↓, IKK↓, NF-κB↓	Ho et al. (2009)
CID-2,353 (Phenolic)					
*Berberis vulgaris*					
Gypenosides (Terpene)	*In vitro:* SAS (tongue OSCC)	Wound-healing assay: 180 μM for 48 h	• Cell migration and invasiveness ↓	• NF-κB↓, COX-2↓, ERK1/2↓, MMP-9↓, MMP-2↓, SOS↓, Ras↓, uPA↓, FAK↓, Akt↓	Lu et al. (2011)
Gynostemma pentaphyllum Makino				• mRNA levels of MMP-2↓, MMP-7↓, MMP-9↓	
*Physalis angulate* (crude extract)	*In vitro:* HSC-3 (OSCC), huvec (human umbilical vein endothelial cells)	Wound-healing assay and Trans well assay: ∼5, 10 μg/ml for 12, 24 h respectively	• Cell migration and invasiveness ↓	• MMP-9 ↓, MMP-2 ↓ and u-PA ↓	Hseu et al. (2011)
				• TIMP-1 ↑, TIMP-2↑, PAI-1 ↑ and PAI-2 ↑	
	*In vivo:* Fertilized chick embryos	CAM assay: 10–20 μg/ml for 48 h	• *In-vivo* angiogenesis ↓	• VEGF ↓	
*Selaginella tamariscina* (crude extract)	*In vitro* study*:*HSC-3 (tongue OSCC)	Scratch-wound assay: ∼75, 50 μg/ml for 12 and 24 h, respectively	• Cell motility ↓	• MMP-2 ↓, MMP-9 ↓	Yang et al. (2013)
				• TIMP-1 ↑, TIMP-2 ↑	
				• MMP-9 promoter activity ↓	
			• Cell migration and invasiveness ↓	• Binding of CREB, SP-1 and AP-1 to the MMP-2 promoter ↓	
				• Akt phosphorylation ↓	
*Selaginella tamariscina* (crude extract)	*In vitro:* HONE-1 (NPSCC)	Scratch-wound assay: >25*μg/mL for 24 h	• Cell motility ↓	• MMP-9 ↓	Hsin et al. (2013)
			• Cell migration and invasiveness ↓	• FAK-src phosphorylation ↓	
				• ERK1/2 phosphorylation ↓	
Phenethyl isothiocyanate	*In vitro:* SAS (tongue OSCC)	Matrigel invasion assay: 0.5, 1, 2 μM for 48 h	• EGF-stimulated invasion↓	• MMP-2↓, MMP-9↓	Chen et al. (2013)
CID-16741 (Alkaloid) *Brassica oleracea var. italica*				• TIMP-1↑, TIMP-2↑	
			• No effect on cell viability	• Activation of EGFR↓	
				• PDK1↓, P13K (P85) ↓, AKT↓, NF-κB↓, MMP-1↓, MMP-2↓	
				• Phosphorylation of p38↑, JNK↑, ERk↑, MAPK signaling pathway↑	
*Galium verum* (crude extract)	*In vitro:* FADU (hypopharyngeal SCC), HLaC78 (LSCC), MK (mucosal keratinocytes)	*In vitro* motility assays: Sub-lethal doses of 33.3 μL/ml	• Cell growth ↓	• MMP-9 ↑, TIMP-1 ↑	Schmidt et al. (2014)
			• Cell migration and invasiveness ↓		
Resveratrol	*In vitro* study*:* SCC-9 (tongue OSCC)	Wound-healing assay: >25 μM for 24 h	• No cytotoxicity	• MMP9↓	Lin et al. (2015)
CID-445154 (Phenolic)			• Cell motility↓	• Phosphorylation of ERK and JNK↓	
*Arachis hypogaea*			• Cell migration and invasiveness ↓	• MAPK activation↓	
Evodiamine	*In vitro:* HONE1, CNE1 (NPSCC)	Wound-healing assay: ∼25 μM for 24 h	• Cell migration and invasiveness ↓	• mRNA and protein of MMP-2↓	Peng et al. (2015)
CID-442088 (Alkaloid)				• Translocation of NF-κB (p65)↓	
*Tetradium* spp.				• MMP-2↓	
				• Phosphorylation ERK1/2↓	
Nobiletin	*In vitro:* HONE-1, NPC-BM (NPSCC)	*In vitro* wound closure: 40 μM for 24 and 48 h	• Cell migration and invasiveness ↓	• MMP-2↓, TIMP-2↑	Chien et al. (2015)
CID-72344 (Phenolic)	*In vivo:* Male BALB/c nude mice		• *In vivo* tumor formation and metastasis↓	• NF-κB and AP-1 signaling pathways↓	
*Citrus reticulata*				• Phosphorylation of ERK1/2↓—	
Lycopene	*In vitro:* FaDu (hypopharyngeal SCC), Cal-27 (tongue OSCC)	Colony formation: 25 μM for 24, 48 and 72 h	• Cell proliferation↓, colony formation↓	• Bcl-2↓, Bax↓, caspase-3↓, cleaved caspase-9↓	Ye et al. (2016)
CID-394156 (Terpenes)			• Cell invasion↓	• Phosphorylation of AKT↓, ERK↓	
*Daucus carota subsp. Sativu* ***s***				• PI3K/AKT, MAPK pathways↓	
*Toona sinensis* (crude extract)	*In vivo:* Male syrian golden hamsters	***In vivo*** treatment: 1 g/kg body weight for 4 weeks	• Incidence of SCC↓, epithelial dysplasia↓	• Proteins of survivin↓, XIAP↓, PCNA↓, iNOS↓, and COX-2 ↓	Wang et al. (2016)
*Toona sinensis*			• Tumor number↓, tumor volume↓, tumor burden↓, severe dysplastic lesions↓		
			• Apoptosis↑		
Triptolide	*In vitro:* CNEn (NPSCC)	Cell clonogenicity: 4 ng/ml with IR at 0, 2, 4 and 8 Gy	• Cell growth↓, colony number↓	• Bax↑	Zhang et al. (2016)
CID-107985 (Terpene)	*In vivo:* BalB/C nude mice female	*In vivo* treatment: 0.075 mg/kg per day	• ionizing radiation↑ induces apoptosis	• Proteins phosph-NF-κb p65↓, Bcl-2↓ and VEGF↓	
*Tripterygium wilfordii*			• Anti-angiogenesis effects		
Raspberries (crude extract)	*In-vitro* study*:* SCC-9, SAS (tongue OSCC)	Scratch-wound assay: ∼100 μg/ml for 48 h	• No cell viability effect	• MMP-2 mRNA↓, protein level ↓ and enzyme activity↓	Huang et al. (2017)
*Rubus idaeus*			• Cell migration and invasiveness ↓	• FAK-src phosphorylation ↓	
			• Metastasis ↓	• ERK1/2 phosphorylation ↓	
Tricetin	*In vitro:* SCC-9, HSC-3 (tongue OSCC), OECM-1 (OSCC)	Boyden chamber assays: >20 µM in 24 h	• Cell migration and invasiveness ↓	• MMP9 enzyme activity↓, MMP9 mRNA expression↓	Chung et al. (2017b)
CID-5281701 (Phenolic)					
*Eucalyptus globulus*				• Regulated MAPK signaling pathway by *p*-JNK1/2↓ and p-p38↓	
				• p38/jnk-MMP9 axis signaling ↓	
Raspberries (crude extract)	*In-vitro* study: HONE-1, NPC-39 and NPC-BM (NPSCC)	Wound-healing assay: 100 μg/ml for 12 and 24 h	• Tumor cell migration ↓	• MMP-2 mRNA↓, protein level ↓ and enzyme activity↓	Hsin et al. (2017)
*Rubus idaeus*			• Invasive ability ↓	• ERK1/2 phosphorylation ↓	
				• Inhibits MAPK signaling pathway	
Quercentin	*In vivo:* Male syrian hamsters	*In vivo* treatment: 50 mg/kg for 14 weeks	• DMBA induced carcinogenesis and apoptosis↓	• NF-Kb p50↓, p65↓	Zhang et al. (2017)
CID-4444051 (Phenolic)			• Tumor incidence↓	• DBMA induced Bcl-2↓, Bax↓	
*Allium cepa*					
*Leucaena leucocephala* (crude extract)	*In-vitro* study*:* SCC-9, SAS (tongue OSCC)	Scratch-wound assay: ∼20 μg/ml for 6–48 h	• Cell motility ↓	• MMP-2 ↓	Chung et al. (2017a)
			• Cell migration and invasiveness ↓	• ERK and p38 phosphorylation ↓	
			• Anti-metastatic activity		
Gallic acid	*In vitro:* NPC-BM1 (NPSCC)	*In Vitro* matrix invasion: 25 µM in 24 h	• Invasion↓	• mRNA expression and transcription of MMP-1↓	Pang et al. (2017)
CID-370				• MMP-1 promoter↓, AP-1↓ and ETS-1↓, TIMP-1↓	
*Hamamelidaceae* spp.				• p38 MAPK pathway ↓	
Salvianolic acid A	*In vitro:* SCC-9, SCC-25 (tongue OSCC)	Wound healing migration assay: 50 µM for 24, 48 h	• Cell migration and invasiveness ↓	• MMP-2↓, p-c-Raf↓, *p*-MEK1/2↓, *p*-ERK1/2↓ protein	Fang et al. (2018)
CID-5281793 (Phenolic)					
*Salvia miltiorrhiza*			• Anti-metastatic		
Bitter melon (crude extract)	*In vivo:* C57BL/6 mice	*In vivo* treatment: 4-NQO- 50 μg/ml; BME- 30% v/v, 600 mg/mouse	• No histological abnormality	• PCNA↓	Sur et al. (2018)
*Momordica charantia*			• Delayed tumor initiation	• GO categories “Keratin filament”↓,“extracellular region”↓, “GTP binding”↓, “extracellular space”↑, “cytokine activity”↑, “immune response”↑, “positive apoptotic process”↑	
			• Incidence of tongue tumor↓		
*Eclipta prostrata* (crude extract)	*In vitro:* SCC-9, HSC-3, (tongue OSCC) TW2.6 (OSCC)	Boyden chamber assay: ∼100 μg/ml for 24 h	• Cell migration and invasiveness ↓	• MMP-2↓	Liao et al. (2018)
*Eclipta prostrata*			• Oral cancer metastasis↓	• Phosphorylated ERK1/2↓	
Black raspberries (crude extract)	*In vivo:* Male F344 mice	*In vivo* treatment: 4-NQO- 20 μg/ml	• Oral lesion incidence and multiplicity↓	• Aldoa↓, Hk2↓, Tpi1↓, Pgam2↓, Pfkl↓, Pkm2↓	Knobloch et al. (2019)
*Rubus occidentalis*		BRB- 5 and 10% w/w for 6 weeks		• PKA-AMPK pathway genes↓	
Pinosylvin	*In vitro:* SAS, SCC-9, HSC-3 (tongue OSCC)	*In vitro* wound closure: ∼20 μM for 2 h	• Cell migration↓	• Enzymatic activity and protein level of MMP-2↓	Chen et al. (2019)
CID-5280457 (Phenolic)					
*Gnetum cleistostachyum*				• TIMP-2↑, phosphorylation of ERK1/2↓	
*Duchesnea indica* (crude extract)	*In vitro* study*:* SCC-9, SCC-14 (tongue OSCC) and TW2.6 (OSCC)	Wound-healing assay: ∼20, 40 μg/ml for 24, 48 h, respectively	• Cell motility ↓	• MMP-2 ↓	Yang et al. (2019)
			• Cell migration and invasiveness ↓	• ERK1/2 phosphorylation ↓	
			• MAPK/ERK signaling pathway ↓	• FAK Y397, src, c-raf, and MEK1/2 phosphorylation ↓	

Abbreviations: 4-NQO: 4-Nitroquinoline 1-oxide; Akt: Protein kinase B; AMPK: AMP-activated protein kinase; AP-1: Activator protein 1; Bax: Bcl-2-associated X protein; Bcl-2: B-cell lymphoma 2; COX-2: Cyclooxygenase-2; c-Raf: c- Rapidly Accelerated Fibrosarcoma; CREB: cAMP response element-binding protein; DMBA: 7,12-dimethylbenz(a)anthracene; ERK: Extracellular-signal-regulated kinase; FAK-Src: Focal adhesion kinase-Steroid receptor coactivator; GO: Gene ontology; HK2: hexokinase 2; IKK: Inhibitor of nuclear factor-κB (IκB) kinase; iNOS: Inducible nitric oxide synthase; JNK: c-Jun N-terminal kinase; LC3: Microtubule-associated protein 1A/1B-light chain three; LSCC: Laryngeal squamous cell carcinoma; MAPK: Mitogen-activated protein kinase; MMP: Matrix metallopeptidase; mTOR: mammalian target of rapamycin; NF-κB: Nuclear factor kappa light chain enhancer of activated B cells; NPSCC: Nasopharyngeal squamous cell carcinoma; OSCC: Oral squamous cell carcinoma; PI3K: Phosphatidylinositol 3-kinase; PAI-1: Plasminogen activator inhibitor-1; PCNA: Proliferating cell nuclear antigen; PDK1: 3-Phosphoinositide-dependent kinase 1; PFK1: Phosphofructokinase-1; PGAM2: Phosphoglycerate mutase 2; PKA: Adiponectin activates protein kinase A; PKM2: Pyruvate kinase M2; MEK: Mitogen-activated protein kinase; SCC: Squamous cell carcinoma; SP-1: Specificity protein 1; TIMP-1: Tissue inhibitor of metallopeptidase inhibitor 1; Tpi1: Triosephosphate isomerase; u-PA: urokinase-type plasminogen activator; XIAP: X-linked inhibitor of apoptosis protein.

*In vitro* studies conducted with epigallocatechin-3-gallate, berberine, gypenosides, phenethyl isothiocyanate, resveratrol, tricetin, nobiletin, evodiamine, salvianolic acid A, gallic acid, pimosylvin, and extracts of *Eclipta prostrata*, *Physalis angulata*, *Selaginella tamariscina*, *Leucaena leucocephala*, *Duchesnea indica*, rasberries (*Rubus idaeus*), and *Galium verum* downregulated the expression of MMPs (Thomas et al., 1999; Ho et al., 2007; Ho et al., 2009; Hseu et al., 2011; Lu et al., 2011; Chen et al., 2013; Chien et al., 2015; Lin et al., 2015; Peng et al., 2015; Chung HH. et al., 2017; Chung TT. et al., 2017; Huang et al., 2017; Pang et al., 2017; Fang et al., 2018; Liao et al., 2018; Chen et al., 2019; Yang et al., 2019). The increase in MMPs is generally associated with invasive and metastatic phenotype of oral carcinoma (Thomas et al., 1999). Tissue inhibitor of metalloproteinases (TIMPs) are endogenous inhibitors of MMPs, and play a role in cell migration and wound healing. TIMPs were found to be up-regulated in phenethyl isothiocyanate, nobiletin, gallic acid, *Physalis angulata*, *Selaginella tamariscina*, and *Galium verum* (Chen et al., 2013; Hsin et al., 2013; Chien et al., 2015; Yu et al., 2016; Pang et al., 2017).

Berberine, phenethyl isothiocyanate, resveratrol, gypenosides, lycopene, evodiamine, gallic acid, nobiletin, tricetin salvianolic acid A, pinosylvin, and extracts of *Eclipta prostrata*, *Selaginella tamariscina*, *Leucaena leucocephala*, *Duchesnea indica*, and *Rubus idaeus* inhibited the MAPK/ERK pathway (Ho et al., 2009; Hseu et al., 2011; Chen et al., 2013; Hsin et al., 2013; Schmidt et al., 2014; Chien et al., 2015; Ye et al., 2016; Yu et al., 2016; Chung HH. et al., 2017; Huang et al., 2017; Pang et al., 2017; Fang et al., 2018; Yang et al., 2019). Additionally, genistein, triptolide, and *Physalis angulata* extract downregulated VEGF expression (Myoung et al., 2003; Hseu et al., 2011; Zhang et al., 2016).

*In vivo* studies with nobiletin on male BALB/c nude mice suppressed tumor formation and metastasis by downregulating NF-κB translocation, MMP-2, and TIMP-2 proteins, and decreased phosphorylation of ERK1/2 (Chien et al., 2015). *Toona sinensis* crude extract decreased the incidences of SCCs, tumor number, tumor volume, and tumor burden in male Syrian golden hamsters by downregulating protein levels of survivin, XIAP, PCNA, iNOS, and COX-2 (Wang et al., 2016). Delayed tumor initiation incidence was reported in bitter melon extract–fed mice (Sur et al., 2018). Oral lesion incidence decreased in 4NQO exposed mice after being fed a black raspberry diet by downregulating PKA-AMPK pathway genes, which regulates mitochondrial functions (Knobloch et al., 2019).

Most of the reported clinical trials focusing on chemoprevention in HNC have been conducted on oral premalignant lesions (Table 7). Historically, clinical studies conducted on HNC chemoprevention with natural agents have centered on the use of retinoid. Bichler et al. (1983) reported that serum levels of retinol, RBP, and PACB were significantly lower in patients with carcinomas of the HN region (Bichler et al., 1983). This was considered to be of significance in tumor development studies, and since then, it has been corroborated by various research groups such as by Kapil et al. (2003). One of the initial studies conducted with retinoids was by Hong et al., 1986. The double-blind study demonstrated the effectiveness of 13-cRA in reducing the size of oral premalignant lesions in 44 patients. In a study conducted by Stich et al. (1988a) on 65 patients having well-developed oral leukoplakia, a complete remission in the lesions was observed in 57.1% of patients in the vitamin A group as compared to 3% of patients in the placebo group (Stich et al., 1988a). An interesting study was also conducted by Mathew et al., (1995) using lyophilized *Spirulina fusiformis*, an effective source of dietary vitamin A and other micronutrients (Mathew et al., 1995). A 1 g/day dose of oral *Spirulina fusiformis* powder demonstrated an effective chemoprevention activity by producing a complete response in 20/44 subjects in the treatment group as compared to 3/43 subjects in the placebo group. A partial response was observed in five patients in the *Spirulina fusiformis* treatment group as compared to zero in the placebo group.

**TABLE 7:** Clinical studies in chemopreventive phytochemicals/herbal derivatives against HNC.

Phyto-chemical/herbal extract/	Type of study	Study participants	Dosage, treatment duration, and follow-up	Criteria (inclusion/exclusion)	Clinical outcome	Adverse effects reported	Ref
13-cis-Retinoic acid	Randomized double-blind trial	• *n* = 44	1–2 mg/(kg of body weight)/day (oral)3 months (6 months)	Inclusion	• decrease in size of lesions in 67% of drug recipients as compared to 10% in placebo recipients	• Toxic effects acceptable except in 2 cases	Hong et al. (1986)
		• Retinoic acid Placebo::24:20		• Patients with histologically confirmed oral premalignant lesions			
					Histologic response in 56% of drug recipients with clinical response	• Cheilitis, facial erythema, peeling of the skin in 79% drug recipients	
		• Age- <50: 6 50-69: 29 > 70: 6		Exclusion			
				• Fertile women			
		• Gender M:F::31:13	Evaluation: every 2–3 weeks (4–6 weeks)	• People taking >25,000 USP/day units of vitamin A	• Dysplasia reversed in 54% of the drug recipients as compared to 10% of placebo recipients	• Conjunctivitis in 54%	
		Risk factors:					
		o Alcohol and tobacco: 20/44		• People diagnosed with oral cancer 2 years before the study			
		o Alcohol only: 11/44			• Histological improvement in 50% of drug recipients	• Hypertriglyceridemia in 71%	
		o Tobacco only: 9/44					
		o Neither: 4/44					
Vitamin A	Randomized double-blind Trial	• *n* = 65	200,000IU of vitamin A/week administered in the form of capsules administered twice weekly (0.14 mg/kg body weight per day)	Inclusion	• Complete remission in 57% in VitA as compared to 3% in placebo	• No adverse effects	Stich et al. (1988a)
		Vitamin A:Placebo::30:35		Betel quid chewers with well-established leukoplakias			
		• Completed study = 54			• No new leukoplakias in all chewers receiving vitamin A, as compared to 21% in the placebo		
		Vitamin A: Placebo::21:33					
		Risk factors:			• Number of layers of spinous cells decreased in 85%		
		o Betel quid chewing and alcohol drinking: 37%	Duration of trial- 6 months				
		o Chewing, drinking and smoking of bidis:28%			• Loss of polarity of basal cells was reduced from 72 to 22%		
		o Chewing and smoking of bidis: 2%	Evaluation		• Subepidermal lymphocytic infiltration diminished from 66.7 to 5.5%		
		• Betel quid chewing without any additional oral habit: 16%	Every 3 months		• Nuclei with condensed chromatin disappeared from the epidermal layer (72.2% before to 0% at the trial end)		
Beta-carotene and beta-carotene plus vitamin A	Randomized double-blind Trial	• *n* = 130	Group I: 180 mg beta-carotene/week	Inclusion	Frequency (%) of micronucleated cells was reduced in leukoplakia of betel quid chewers	• No adverse effects	Stich et al. (1988b)
		Three groups receiving:		Betel quid chewers with well-established leukoplakias			
		o Beta-carotene: Group I, 35 people					
		o Beta-carotene and vitamin A: Group II, 60 people			% micronucleated cells placebo, leukoplakia		
		o Placebo-group III, 35 people			o Before: 3.69 ± 1.22		
		• Completed study: Groups I, II, III are: 30, 54, 26	Group II: 180 mg beta-carotene + 100,000 IU vitamin A/week		o After: 4.00 ± 1.32		
		• Age: 48.8 ± 12.9			• % micronucleated cells beta carotrene, leukoplakia		
		Risk factors:			o Before: 4.09 ± 1.10		
		o Betel quid chewing and alcohol drinking: 37%			o After: 1.18 ± 0.77		
		o Chewing, drinking and smoking of bidis: 28%	Group III: Placebo For 6 months		% micronucleated cells beta-carotene + vitamin A, leukoplakia		
		o Chewing and smoking of bidis: 2%			o Before: 4.01 ± 1.05		
		• Betel quid chewing without any additional oral habit: 16%			o After: 1.16 ± 0.94		
					• Well-established leukoplakias regressed. Remission of leukoplakias in		
					o Group I: 14.8%		
					o Group II: 27.5%		
					o Group III: 3%		
					• The development of new leukoplakias was inhibited new leukoplakia occurrence:		
					o Group I: 14.8%		
					o Group II: 7.8%		
					o Group III: 21.2%		
Beta-carotene		• *n* = 25	30 mg/day for 3 months	Inclusion	• 17 had major responses (two complete, 15 partial), a response rate of 71%	• No significant toxicity attributable to beta-carotene was encountered in this trial	Garewal et al. (1990)
		• completed study = 24		• Adult patients diagnosed as having clinically measurable oral leukoplakia while undergoing routine dental examinations			
		• Gender Bias- M:F::21:3	Responding patients: 3 more months (rest were taken off the therapy)				
		• Age (years)					
		o < 50: 9		Exclusion	• Relapses after discontinuation of treatment: 8/11 responders within 3 months of cessation of drug		
		o 50-69: 9		Patients taking high daily doses of vitamin A			
		o > 70: 6					
		Risk factors	Evaluation: 2–3 months intervals				
		o Alcohol and tobacco: 9					
		o Alcohol only: 7					
		o Tobacco only: 4					
Isotretinoin	Randomized placebo-controlled study	• *n =* 103	50–100 mg per square meter of body-surface area per day	Inclusion	• No significant differences between the two groups in the number of local, regional, or distant recurrences of the primary cancers	• Mild or moderate	Hong et al. (1990)
		• Completed study = 100		• Clinically free of disease after having undergone surgery or radiation therapy (or both) for histologically confirmed primary OSCC, OPSCC, HPSCC, or LSCC			
		• Drug:Placebo::49:51	For 12 months and follow-up for 32 months	Exclusion	• The isotretinoin group had significantly fewer second primary tumors	• Severe skin dryness, cheilitis, hypertriglyceridemia, and conjunctivitis, occurred in 12, 2, 6, and 8%, respectively, of isotretinoin recipients	
		• Gender bias: M:F::78:32		• Abnormal renal or hepatic function			
		• Age- 31–73 years		• Distant metastasis or a Karnofsky performance score <60%			
		Risk factors –		• Previous chemotherapy, within the 2 years	• Only 2 patients (4 percent) in the isotretinoin group had second primary tumors, as compared with 12 (24%) in the placebo group		
		• Smoker:	Evaluation:	• A diagnosis of any cancer except *in situ* or T1 HNSCC or skin cancer other than melanoma			
		o Current:Former::33:57	Monthly during treatment and once in three months during follow-up	• Women of reproductive capacity	• Multiple second primary tumors occurred in four patients, all of whom were in the placebo group		
		• Alcohol		• Patients taking large doses of vitamin a (>25,000 USP units per day)			
		o Yes:No::55:45					
Alpha-tocopherol	A single-arm phase II study	• n = 43	400 IU twice daily for 24 weeks	Inclusion	• Clinical response (complete or partial) in 20/43 patients was observed	• No grade 3 or 4 toxic effects were reported	Benner et al. (1993)
		• Gender bias- M:F::24:19		Patients with bi-dimensionally measurable symptomatic leukoplakia (i.e., lesions associated with discomfort such as burning or pain) or leukoplakia with dysplasia			
		• Mean age: 55.6 years					
		Tobacco use				• Observed side effects were grade 1: *Vertigo*, extremity aches, nausea, diarrhea, intestinal cramps, breast enlargement, fatigue, and fluid retention	
		o Current: 48.8%					
		o Past: 30.3%			• Histological response was observable in 9 patients		
		o Never 20.9%	Evaluation: At 6, 12, and 24 weeks				
		• Alcohol use					
		o Current: 55.8%					
		o Past: 18.6%					
		• Never: 25.6%					
Isotretinoin Beta-carotene	Uncontrolled open trial conducted in two phases	**Phase I** (induction therapy with high dose isotretinoin) *n* = 70	**Phase I**: isotretinoin: 1.5 mg/kg body weight/day	Inclusion	**Phase I**	**Phase I**: Substantial side effects. 23 patients with grade 3 or 4 toxic reactions. Dry skin, cheilitis, conjunctivitis, Triglycerdemia	Lippman et al. (1993)
				• Oral lesions that were histologically confirmed as pre-malignant and could be measured in two dimensions	• Partial or complete response was observed in 55% of the patients and stable disease maintained in 35% of the patients		
		Completed/evaluated: 66	**Phase II**: Low dose isotretinoin: 0.5 mg/kg body weight/day	• Normal hepatic and renal functions acceptable	**Phase II**		
		**Phase II**: (Maintenance therapy with low dose isotretinoin OR beta-carotene with patients who responded to induction therapy) *n* = 59	Beta-carotene: 30 mg/day	• Acceptable fasting triglyceride levels at entry	• Positive outcome (improved/stable lesions) was observed in 92 percent (22) patients on low dose isotretinoin therapy		
		Completed/evaluated: 53	**Phase I**: 3 months	Exclusion		**Phase II:** Relatively mild, favoring the beta-carotene group. Hypertriglycerdemia, mild and reversible skin yellowing, dry skin, cheilitis, conjunctivitis	
		• Beta-	**Phase II**: 9 months	• High current vitamin A intake (>25,000 USP units per day)	• 45 percent (13) patients on beta carotene therapy showed a positive response		
		Carotene:isotretinoin::33:26	Evaluation: Every 4 weeks	• High beta-carotene intake			
				• History of oral cancer within two years before study			
Riboflavin, retinol vitamin E, and beta-carotene	Randomized double blind trial	• *n* = 532	Riboflavin (R): 80 mg/week	**Inclusion:**	• Significant decrease in the prevalence odds ratio (OR) of oral leukoplakia was observed after 6 months of treatment	Nausea, vomiting, and itching	Zaridze et al. (1993)
		• Completed study 487 at 6 months 471 at 20 months	Retinol (VA): 100,000 IU/week	Only men who had a diagnosis of chronic esophagitis and/or oral leukoplakia			
			Beta-carotene (BC): 40 g/day				
		• Gender bias: All men	Vitamin E (VE): 80 mg/week				
		• Age: 50–69 years	In 4 groups with placebo (*p*)				
		• 191 with leukoplakia	1: *p*,*p*,*p*				
			2: R,*p*,*p*				
			3: *p*,VA,VE,BC				
			4: R,VA,VE,BC				
			For 20 Months Evaluation: At 6th and 20th month				
Beta-carotene, ascorbic acid, and alpha-tocopherol	Open Trial	• *n* = 7	Beta-carotene: 30 mg/day	Inclusion	• Clinical improvement of the oral lesion was noted in 55.7% of patients	No side effects were reported	Kaugars et al. (1994)
		• Gender bias M:F::45:34	Ascorbic acid: 1,000 mg/day	• Patients with clinically apparent oral leukoplakic lesions that had been histologically verified as either hyperkeratosis or epithelial dysplasia with hyperkeratosis	• 22 (48.8%) of 45 patients who continued their pre-study levels of risk-factor exposure had clinical improvement		
		• Age 25–85 years	Alpha-tocopherol: 800 IU/day	Exclusion:			
		• Risk factors (smoking, smokeless tobacco, alcohol): 65/79	For 9 months Evaluation: At 1st, 3rd, 6th, 9th month	Cases that were either consistent with lichen planus or were suggestive of lichenoid change	• 4 out of 9 patients who had never used either tobacco or alcohol showed clinical improvement		
		• Risk factor reduction or cessation during the course of the study: 20/65					
*Spirulina fusiformis* (lyophilized powder)	Blind Placebo Controlled trial	• n = 115 SF:Placebo::60:55	Lyophilized: *Spirulina fusiformis* 1 g/da for 12 months with a 2 years follow-up evaluation: Every 2 months during supplementation	**Inclusion**:	• 20/44 subjects in the SF group showed a complete response as compared to 3/43 subjects in the placebo group	Headache, muscular pain	Mathew et al. (1995)
		• Completed study = 87 SF:Placebo::44:43		Subjects with oral leukoplakia	• 5 subjects in SF group showed a partial response as compared to 0 in the placebo		
		• Mean age: 47.1 years			• The CR rates were 46% (17 of 37) for lesions 2 cm in diameter • After 1 year of stopping supplementation		
		• Smokers-Yes:No::28:59					
		• Alcohol-Yes::No::52:35			9 of 20 (45%) subjects with CR in the SF arm reported with recurrence of lesions		
		• Chewers-Yes:No::84:87			• 2 years follow-up: malignant transformations were observed in 10% of subjects in the placebo group and 5% of subjects in SF group		
Retinyl palmitate	Randomized Blind Trial	• *n* = 106	200,000 IU per week (administered orally) for 1 year with 2 years follow-up	**Inclusion**	• No second primaries were observed in subjects in the vitamin a group	No clinically obvious side effects (dryness of the tongue in two subjects)	Jyothirmayi et al. (1996)
		• Drug:Placebo::56:50		• Patients with HNC with complete clinical regression of lesions on follow-up after therapy			
		• Gender bias: M:F::73:33		• Have had either radical radiotherapy or surgery or both			
		• Completed study = 93					
		• RP:Placebo::50:43	**Evaluation:** Every 2 months during supplementation	**Exclusion**	• 2 subjects developed primaries (1 case of tongue cancer and 1 of floor of mouth cancer) in the placebo group		
		Risk factors		• Patients with clinical evidence of disease, abnormal kidney and liver function			
		o Chewing tobacco:65					
		o Smoking: 61					
Retinyl palmitate	Open trial conducted in two phases	• *n* = 20	**Phase I**	Inclusion	• Complete remission rate observed in 75% (15 of 20 patients)	None of the patients had more than grade 2 reactions; grade 3 and 4 reactions or a withdrawal because of intolerable toxic effects were not observed	Issing et al. (1996)
		• Gender bias-M:F::17:3	300,000 IU/day to 1,500,000 IU/day in patients showing resistant lesions in the fifth week	• Presence of larynx leukoplakia which could be measured in two dimensions			
		• Age range: 46-80		• Normal renal and hepatic function			
		• Risk factors:		• Acceptable fasting triglyceride levels upon entry			
		o Alcohol and tobacco: 7	**Phase II**	Exclusion	Partial response was observed in 5 patients. Among the 5 patients with partial response, 3 relapsed.		
		o Alcohol only: 8; tobacco only: 1	150,000 IU/day for patients who responded to therapy	• Possibility of pregnancy			
		• Neither: 4	Median duration of treatment and follow-up: 18 months	A current intake of large doses of vitamin a (>25,000 USP units per day) or betacarotene			
			**Evaluation:** Every 4 weeks during study and 3 months during follow-up				
Vitamin a and beta-carotene	Randomized double blind trial	• *n* = 160	Vitamin A: 300,000 IU/week	NIL	• Vitamin a group: Complete regression in 22 of 42 (52%) subjects	Headache, muscular pain, dry mouth	Sankaranarayanan et al. (1997)
		• Completed study = 131					
		• Vitamin A:Beta-Carotene:Placebo::50:55:55	Beta-carotene:360 mg/week		• Beta-carotene group: 15/46 (32%) of subjects showed complete regression		
		• Completed study: vitamin A: Beta-carotene:: 42:46:43	For 12 months and 1 year follow-up				
		• Gender bias-M:F::1.79:1	**Evaluation:** Every 2 months during supplementation		• Homogeneous leukoplakias and smaller lesions responded better than non-homogeneous and larger lesions		
		• Chewing tobacco-Yes:No::127:4			• 1 year after stopping treatment:		
		• Smokers-Yes:No::41:70			o 64% complete responders with vitamin a and 53% complete responders with beta carotene developed recurrent lesions and		
		• Alcohol-Yes:No::72:59			• 10% subjects in the placebo group and 5% in the beta carotene group developed malignancy at the site of leukoplakia		
Tea	Double-blind intervention trial	*n* = 64	3 g, 4:1:1 mixture of green tea, green tea polyphenols and tea pigment administered orally and applied topically	Inclusion:	• 37.9% showed decrease in the lesions among 29 treated patients; 3.4% showed an increase		Li et al. (1999)
		Tea:Placebo::32:32		Patients suffering from oral leukoplakia			
		Age-23–28 years			• 10% of patients in the placebo group showed an decrease in lesions; 6.7% showed an increase		
		Gender bias-M:F::40:24					
		Smokers	For 6 months Evaluation: Every 2 months		• Micronucleated exfoliated oral mucosa cells in treated group was lower in placebo group		
		Yes:No::46:18					
		Evaluated/completed trial: 59					
Interferon-alfa, 13- cis -retinoic acid, and alpha-tocopherol	Phase II single-arm trial	• n = 45	Dose modifications based on monthly evaluated toleration of therapy	Inclusion	At median 24-months of follow-up, the clinical end point rates were	Mild to moderate mucocutaneous side effects, flu-like symptoms, anorexia, and weight loss, fatigue, peripheral neuropath optic neuritis, mild to moderate hypertriglyceridemia	Shin et al. (2001), Seixas-Silva et al. (2005)
		• Gender bias-M:F::36:9	Interferon-alfa: (1-3)X106 IU/m2 subcutaneous injection, three times a week	• Confirmed diagnosis of squamous cell carcinoma of the oral cavity, oropharynx, larynx, or hypopharynx	Among 45		
		• Age	13-cRA: 20–50 mg/m2/day	• Locally advanced stage III or IV disease	• 9% for local/regional recurrence (four patients)		
		o Median: 52 years	Alpha-tocopherol: 1,200 IU/day	• Enrolled a minimum of 3 weeks and maximum of 24 weeks after definitive local therapy with surgery, radiotherapy, or both	• 5% for local/regional recurrence and distant metastases (two patients)		
		o Range: 43–70 years		• Should not have received chemotherapy, immunotherapy, or hormonal therapy before entry onto the study	• 2% for SPT (one patient), which was acute promyelocytic leukemia		
		• Initial stage	For 12 months	• Must have recovered from the acute toxic effects of surgery, radiotherapy, or both	• Median 1- and 2-years rates of overall survival were 98 and 91%, respectively, and of disease-free survival were 91 and 84%, respectively		
		o Stage III: 11		• Must be able to swallow the pills without breaking them	At median 49.4-months of follow-up		
		o Stage IV: 34	**Evaluation:** Every 3 months (monthly check ups for dose modification)	• Life expectancy of >12 weeks	• 9 (20%) of 44 patients experienced progressive disease, 3 since the last report		
		• Prior treatment		• Karnofsky performance status rating of >80%	• Two patients had local recurrences		
		o Surgery: 3		• Adequate bone marrow function and adequate renal and hepatic functions	• 1 had local and distant relapse. The progression-free survival percentages at 1 year, 3 years, and 5 years were 88.9, 82.2 and 80% respectively		
		o Radiotherapy:15	Follow up: median 24 months and median 49.4 months	**Exclusion**	• The overall survival percentages at 1 year, 3 years, and 5 years were 97.8, 88.9 and 81.3% respectively		
		• Surgery and radiotherapy: 27		• If taking megadoses of vitamin a (>25,000 IU)			
				• If they were women of child-bearing potential who were not practicing adequate birth control			
				If they had a baseline triglyceride level > twice the normal range			
ZengShengPing (*Sophra tonkinensis, Polygonum bistorta, Prunella vulgaris*, *Sonchus brachyotus*, *Dictamnus dasycarpus*, and *Dioscorea bulbifer*)	Randomized placebo-controlled trial	*n* = 120	3.6 g per day for 8–12 months	Inclusion	Positive response was observed in 67.8% (40/59) patients of ZSP group, and in 17% (9/53) patients of placebo group		Sun et al. (2010)
		Completed/evaluated = 112		• 20 patients with oral leukoplakia i.e white patch or plaque that cannot be characterized clinically or pathologically as any other disease			
		ZSP:Placebo::59:53					
		Mean age: (ZSP:Placebo)- 52.9:44.4					
		Gender bias-M:F::69:43		Exclusion			
		Risk factors-		• Those with a previous diagnosis of head and neck or oral cancer			
		smokers		• Those currently treated by other drugs or having drug hypersensitivity			
		Yes:No::53:59		• Those requiring extensive dental procedures			
		Drinkers		Those with a history of social or psychiatric situations interfering with study compliance			
		Yes:No::10:102					
Lycopene	Randomized placebo-controlled trial	*n* = 58	Group B: 4 mg/day	Inclusion	• Clinically the patients in groups A, B, C had a mean response of 80, 66.25 and 12.5% respectively		Singh et al. (2004)
		Gender bias-M:F::44:14	Group C: Placebo	• Patients suffering from oral leukoplakia	• Group A:		
		Age: 10–70 years (70% between 31 and 70 years)	For 3 months		o Complete response-11		
			Follow-up: 2 months		o Partial response-7		
			Evaluation: Every 7–10 days during treatment and 15 days during follow-up		o Stable response-2		
					• Group B:		
					o Complete response -5		
					o Partial response-7		
					o Stable response-2		
					• Group C:		
					o Complete response-0		
					o Partial response-3		
					o Stable response-15		
Green tea extract	Phase II, randomized double-blinded placebo-controlled trial	*n* = 41	Group 1	Inclusion	• The clinical response rate was higher in the three combined GTE arms (50%) vs. placebo (18.2%)	Treatment-related adverse events reported by 28 of the 30 (93.3%) patients who received GTE	Tsao et al. (2009)
		Placebo:Group1:Group2:Group3::1:11:9:10	GTE: 500 mg/m2	• Presence of one or more histologically confirmed, bidimensionally measurable OPLs that could be sampled by biopsy and had at least one of the following high-risk features of malignant transformation			
		Gender bias-M:F::19:22	Group 2	• Harboring at least mild dysplasia	• Histologic response rate 21.4% (GTE arms) vs. 9.1% (placebo)	There were only three grade 3 adverse events and no grade 4 or 5 adverse events	
		Smoker	GTE: 750 mg/m2	• Located in a high-risk area (i.e., floor of mouth, ventrolateral tongue, and soft palate)			
		Never: 15	Group 3	• Significant extent of OPL tissue involvement			
		Former: 22	GTE: 1,000 mg/m2	• Presence of symptoms (pain or substantial discomfort)			
		Current: 4	Group 4: Placebo	• Age between ≥18 and ≤75 years	• The clinical response rate was dose dependent—58% in the combined higher-dose GTE arms (group 2 and 3) vs. 36.4% group 1 and 18.2% (placebo)	Common grade 1 and 2 events: Headaches, insomnia, nausea, nervousness, flatulence, gastric reflux, back pain	
		Cigar	For 12 weeks	• Zubrod performance status of <2			
		Former 1	Follow up: 27.5 (median time)	• Adequate hematologic, liver, and renal function			
		Current 3	Evaluation: After 4 weeks	• Adequate cardiac function			
		Smokeless tobacco		• Negative pregnancy test in females of childbearing potential within 7 days before first dose of study medication; use of effective contraceptive method while on the trial			
		Former: 1		Exclusion	• Dose dependency was not seen in histologic response		
		Current: 0		• Known hypersensitivity to oral GTE or its analogs			
		Alcohol		• Use of prior investigational agents within 30 days			
		Never: 8		• Any serious intercurrent illness	• With a median follow-up time of 27.5 months, 15 patients subsequently developed oral cancer with a median time to oral cancer development of 46.4 months		
		Former: 9		• History of prior malignancy with less than a 1-year disease-free interval before study entry			
		Current: 24		• Lactating females patients who were not able to abstain from the consumption of methylxanthine-containing products (including coffee, tea, chocolate, caffeinated soft drinks, and theophylline) and decaffeinated tea			
Black raspberries		*n =* 40	10% w/w	Inclusion criteria	• 16 of the 21 BRB treated lesions decreased in size for an average overall size decrease of 26%	No adverse effects	Mallery et al. (2014)
		BRB:Placebo::22:18	For 12 weeks	• Microscopically confirmed premalignant oral epithelial lesions			
		Gender Bias-M:F::14:24	Follow-up: 3 months	• No use of tobacco products for six weeks prior and during the three-month study	• 17 of the 19 placebo gel treated lesions increased in clinical lesional size with an average increase of 18%		
		Age-32–78 years	Long-term follow-up: 3–31 months	• No previous history of cancer	• 2 BRB gel patients had 100% lesional resolution		
		Smoker		Exclusion criteria	• Statistical decrease in histopathologic grade while placebo gel application did not significantly impact histopathologic grade		
		Y:N::16:24		• Previous or current history of non-basal cell cancer			
				• Use of tobacco products	• After 3 months 6 of 22 BRB and 7 of 17 placebo patients had visible evidence of lesional recurrence at the former treatment sites		
				Either a microscopic diagnosis of no premalignant change or oral squamous cell carcinoma (OSCC) in the pretrial biopsy			
Curcumin	Randomized double blind phase IIB trial	• *n* = 223	Three 600 mg capsules taken twice daily; orally and after food (3.6 g/day) for six months	Inclusion	• Statistically significant difference observed	Moderate/severe AEs were recorded in 4 patients in the curcumin arm including anemia skin/subcutaneous tissue disorders, and hypertension	Kuriakose et al. (2016)
		Drug:Placebo::111:112		• The presence of clinical and histologically confirmed oral leukoplakia >15 mm^2^ in area	• Clinical response: 75 subjects (67.5%) in curcumin arm and in 62 subjects (55.3%) in placebo arm		
		• Gender Bias-M:F::161:62		• 1 cm linearly	• Thirty (27%) subjects in curcumin arm and 46 (41.1%) subjects in placebo arm were non-responders		
		• Smoking-	103 subjects for 6 more months. (Drug:Placebo::53:50)	• No previous biopsy or treatment for head and neck cancer prior to 3 months of accrual	• Histologic response was observed in 25 (22.5%) subjects in the curcumin arm and in 23 (20.5%) subjects in the placebo arm		
		current(C)/Former(F)		• No chemopreventive treatment prior to 3 months of accrual	• Combined (clinical and histological) response was noticed in 65 (58.6%) subjects in the curcumin arm and 50 (44.6%) subjects in the placebo arm		
		Yes:No::112:111 (C:F::61:27)		• Zubrod performance of 0–2	• Curcumin group: Of the 18 subjects available at follow-up, 16 (88.9%) continue to have CR at 12 months		
		• Alcohol- Daily/Non-Daily (D/ND)		• Normal hematological and biochemical parameters			
		Yes:No::93:130 (D:ND::16::51)	**Evaluation:** Monthly during supplementation	Exclusion:	• Placebo arm: 7 of 8 subjects (87.5%) demonstrating no relapse after 6 months follow-up		
		• Chewing Tobacco-Current(C)/Former(F)		The presence of oral submucous fibrosis			
		Yes:No::158:63 (C:F::95:63)					
*Calendula officinalis* and lycopene	Double-blinded comparative study	*n* = 60	Calendula officinalis gel (group 1): 2 mg by weight/g	Inclusion	• Group 1		Singh and Bagewadi, (2017)
		CO:Lycopene::30:30	Lycopene gel (group 2): 2 mg by weight/g	• Patients with clinically and histopathologically confirmed cases of homogeneous leukoplakia	° Average size of leukoplakia before treatment: 4.14 cm2 (standard deviation [SD] = 2.07)		
		Gender bias-M:F::50:10	Administered topically thrice a day		o Average size treatment: 2.09 cm2 (SD = 2.59)		
		Age	For 1 month		Group 2		
		26–75 years (maximum in 56–65 years)	Follow-up: 3 months		° The average size of leukoplakia before treatment: 4.46 cm2 (SD = 2.41)		
					o Average size after treatment: 2.89 cm2 (SD = 3.07)		
					• The mean difference in the reduction in size before and after treatment for group I was 2.0 ± 1.0 cm while for the group II, it was 1.57 ± 0.87 cm		

A study by Stich et al. (1988b) reported on the combined effect of beta-carotene and vitamin A on betel quid chewers in India with well-established leukoplakias. Remission in the group receiving combined treatment was 27.5% as compared to 14.8 and 3% in groups receiving just beta-carotene and the placebo, respectively. The rate of new leukoplakia occurrence was also found to be higher in the beta-carotene (14.8%) and placebo groups (21.2%) that that of new leukoplakia occurrence in the group treated with both beta-carotene and vitamin A (7.8%) (Stich et al., 1988a). The effectiveness of beta-carotene as a chemopreventive agent was also established by Garewal et al., (1990), who in study with 25 patients achieved a response rate of 71% in the group treated with 30 mg/day beta-carotene (Garewal et al., 1990). A comparative study conducted in two phases with beta-carotene and isotretinoin by Lippman et al. (1993) reported that low-dose isotretinoin therapy was significantly more active against leukoplakia than beta carotene when preceded by high-dose induction therapy (Lippman et al., 1993). In another three-arm double-blind study conducted with 160 patients by Sankaranarayana et al. (1997), the vitamin A and beta-carotene arms attained a complete regression of leukoplakia lesions in 52 and 32% of the subjects, respectively, as compared to just 10% in the placebo arm (Sankaranarayanan et al., 1997).

Two clinical studies conducted with retinyl palmitate by Jyothimayi et al. (1996) and Issing et al. (1996) reported a complete inhibition of the formation of secondary primary tumors (SPTs) and a complete remission of leukoplakia lesions in 75% of participants in drug-receiving arms, respectively (Issing et al., 1996; Jyothirmayi et al., 1996). Significant decrease in the prevalence odds ratio of oral leukoplakia was observed by Zaridze et al. (1993) in a double-blind trial conducted among 532 subjects with various combinations of riboflavin, retinol, vitamin E, and beta-carotene (Zaridze et al., 1993). Another combinatorial study with beta-carotene, ascorbic acid, and alpha-tocopherol by Kaugars et al. (1994) noted a clinical improvement in 55.7% of the participants; 48.8% of people who continued their pre-study levels of risk factor exposure showed improvement (Kaugars et al., 1994).

A 24-week study by Benner et al. (1993) using alpha-tocopherol as a single agent to treat patients with oral leukoplakia attained a clinical response in 20 patients from the 43 patients who had signed up for it (Benner et al., 1993). Alpha-tocopherol was part of yet another study by Shin et al. (2001), when delivered with IFN-α and 13-cis-retinoic acid; among 44 patients evaluable at a median 24-month follow-up, 9% had locoregional recurrence, 5% had both locoregional recurrence and distant metastases, and 2% developed an SPT. The overall survival rate at the 24-month follow-up was noted to be 91% (Shin et al., 2001).

Green tea, a widely consumed beverage, has been previously reported to exhibit chemopreventive properties against cancer (Imai et al., 1997). Since it inhibits tumor development, is nontoxic, and is easily available to the general population, it has been a subject of interest in cancer studies. Two clinical studies where green tea was used as an agent to treat precancerous lesions like leukoplakia were included. Li et al. (1999) reported a decrease in lesions in 37.9% patients in the tea-receiving arm as compared to improvement in lesions of only 10% patients in the placebo arm (Li et al., 1999). Tsao et al. (2009) reported a dose-dependent clinical response by randomizing 41 patients in three green tea extract–receiving arms (dosage: 500 mg/m^2^, 750 mg/m^2^, 1,000 mg/m^2^) and 1 placebo arm, with a clinical response in 50% of patients in the three combined arms and a 58% clinical response rate in the two combined higher dose arms (Tsao et al., 2009). They also reported a histological improvement in lesions after treatment.

Lycopene is a carotenoid that is abundant in a human diet and has been associated with a reduced risk of cancer of the upper digestive tract (De Stefani et al., 2000). Singh et al. (2004) reported a dose-dependent response of oral leukoplakia for administration of lycopene, with clinical improvement observed in 80% of patients receiving 8 mg/day lycopene; 66.3% patients receiving 4 mg/day dose showed a clinical response (Singh et al., 2004). A clinical study with lycopene and *Calendula officinalis* by Singh and Bagewadi (2017) reported a reduction in the average size of lesions posttreatment. The mean difference in the reduction in size before and after treatment for Group I was 2% ± 1.0 cm, while for the Group II, it was 1.6% ± 0.9 cm (Singh and Bagewadi, 2017).

Curcumin, a flavonoid derived from *Curcuma longa*, has been extensively investigated for its pharmacological properties. It is known to have antioxidant, anti-inflammatory, and anticancer properties, and thus is a promising phytochemical for HN region chemoprevention. A randomized double-blind phase IIB study by Kuriakose et al. (2016) on 223 patients with oral leukoplakia reported a clinical response in 67.5% of patients in the curcumin arm (dosage: 3.6 g/day for 6 months) and a histological response in 22.5% of patients (Kuriakose et al., 2016).

Sun et al. (2010) conducted a randomized placebo-controlled study with ZengShengPing; a mixture of six medicinal herbs was known to have pharmacological effects. 3.6 g of ZSP administered daily for 8–12 months was observed to produce a positive response in 67.8% of patients in the treatment arm as compared to 17% in the placebo group (Sun et al., 2010).

Mallery et al. (2014) conducted a placebo-controlled clinical trial using topically applied 10% w/w black raspberry (BRB) gel among 40 patients with oral premalignant lesions. The study reported an average decrease of 26% in the size of BRB-treated lesions as compared to an increase in size by 18% in the placebo gel–applied lesions. Two patients in the BRB arm exhibited a complete lesional resolution as compared to zero in the placebo gel group (Mallery et al., 2014).

Although a large volume of data reflects targeting of key pro-carcinogenic signaling pathways by various phytochemicals, none of them directly address their possible impact on HPV infection or in HPV-positive HNC lesions. Therefore, we specifically looked for evidences where phytochemicals have been tested against HNC cells with HPV-positive background.

### Chemotherapeutic and Chemopreventive Phytochemicals/Herbal Derivative With Anti-Cancer and/or Anti-Human Papillomavirus Activity in Head and Neck Cancer

Most of the studies described earlier lack specificity against HPV infection. The natural derivatives having both anti-HPV and anti-HNC activity hold great potential as chemotherapeutic and chemopreventive agents for HNC caused by HPV. However, there are only limited resources in terms of HPV-related HNC model systems. Unlike many other infections, HPV cannot be propagated in *in vitro* cultures or in animal models. Unfortunately, suitable animal models that mimic HPV-driven HNC do not exist. In such a scenario, HPV-positive HNSCC cell lines serve as a suitable *in vitro* system. There are currently only a limited set of HPV-driven HN cancer cell lines developed by different investigators (Table 8). As of now, we could identify only 11 cell lines that have been described as HPV positive, and their HPV genotype has been confirmed. A majority of them have HPV16 positivity, and the genome was found to be integrated (Steenbergen et al., 1995; Ballo et al., 1999; White et al., 2007; Brenner et al., 2010; Ye et al., 2011; Tang et al., 2012; Kalu et al., 2017). Similarly, one cell line each of HPV18 and HPV33 has been reported (Owen et al., 2016; Kalu et al., 2017). Although there are various HNSCC cell lines described so far, their HPV status must be ascertained. These cells lines proved to be useful model systems as they showed p16 positivity and demonstrated higher radiosensitivity (Rieckmann et al., 2013). In these cell line integration of HPV from E1, E2, L1, L2, and LCR have been observed which recapitulate observation in primary tumors by whole genome sequence which suggests various hotspots for HPV integration events in HPV-positive tumors and that may play varied role in the development of HNC (Gao et al., 2019). These cell lines and tumor tissues showed the presence of the viral infection by the presence of viral DNA and transcripts which emerged as valuable tools (Steenbergen et al., 1995; Ballo et al., 1999; White et al., 2007; Brenner et al., 2010; Ye et al., 2011; Tang et al., 2012; Kalu et al., 2017).

**TABLE 8:** List of HPV positive Head and neck cancer cell lines developed and described with their key characteristics.

Cell lines	Anatomical site	HPV type	Key characteristics	Copy number	Developed by	References
93-VU-147T	Oral cavity	HPV16	Three integrated sites	NA	VU University Medical Center, Amsterdam Netherlands	Steenbergen et al. (1995)
			E1-17q21			
			E2-5p15.33 (promoter of TERT gene)			
			L2-3p21 (Intergenic)			
UD-SCC-2	Hypopharynx	HPV16	Two integrated sites	14—23 copies	University of Dusseldorf, Dusseldorf, Germany	Ballo et al. (1999)
			E1-17q12 (Intergenic)			
			E2-1p32.3 (Intron 14 of JAK1 gene)			
UPCI-SCC-90(SCC90)	Tongue	HPV16	Two integrated sites	100—500 copies	University of Pittsburgh, Pittsburgh PA	White et al. (2007)
			E1-12p13 (Intron 1 of ETV gene)			
			E1-9q31.1 (Intergenic)			
UPCI-SCC-154	Tongue	HPV16	Four integrated sites	NA	University of Pittsburgh, Pittsburgh PA	White et al. (2007)
			E1-21p11.1 (Intergenic)			
			E1-11q22-23 (Intron 3 of PGR gene)			
			E2-2q33.2 (exon 14 of TMEM237 gene)			
			E2-7q36 (Intron 3 of PTPRN2 gene)			
UPCI-SCC-152	Hypopharynx	HPV16	Four integrated sites	NA	University of Pittsburgh, Pittsburgh PA	White et al. (2007)
			E1-9q31.1 (Intergenic)			
			E1-12p13 (Intron 1 of ETV gene)			
			E2-9q22.33 (Intergenic)			
			LCR-3q23 (Intron 36 of ATR gene)			
UM-SCC-47	Oral cavity/Tongue	HPV16	Two integrated sites	21—47 copies	University of Michigan, Ann Arbor, MI	Brenner et al. (2010)
			E2-3q28 (Intron 10 of TP63 gene)			
			E2-3q28 (exon 14 of TP63 gene)			
HB-2	Oral mucosa	HPV16	NA	NA	Tong University, Shanghai, China	Ye et al. (2011)
UM-SCC104	Oral cavity	HPV16	Three integrated sites	NA	University of Michigan, Ann Arbor, MI	Tang et al. (2012)
			E1-18q21.3 (Intron 1 of DCC gene)			
			E2-17q22 (Intergenic)			
			E2-17p11.2 (Intergenic)			
UM-SCC-105	Larynx	HPV18	Two integrated sites	NA	University of Michigan, Ann Arbor, MIs	Owen et al. (2016)
			L1-8q12.3/4p15.33 (Intergenic)			
			L1-17q12 (Intergenic)			
HMS001	Oral cavity	HPV16	NA	NA	NA	Kalu et al. (2017)
UT-SCC-45	Floor of the mouth	HPV33	NA	NA	University of Turku, SF-20520 Turku, Finland	Kalu et al. (2017)

Abbreviation: NA, not available.

A limited set of studies have been conducted to examine anti-HPV and anticancer activities in HNC (Table 9). The evidence suggests that HPV-positive cells can serve as suitable tools for screening of anti-HPV and anti-HNC. Green tea polyphenol (-)-epigallocatechin-3-gallate (EGCG), a green tea derivative, exhibits various chemopreventive effects, including inhibition of growth factor–mediated proliferation (Liang et al., 1997; Liang et al., 1999a), induction of G1 arrest (Khafif et al., 1998; Liang et al., 1999b; Liberto and Cobrinik, 2000), and apoptosis (Ahmad et al., 1997; Paschka et al., 1998; Yang et al., 1998; Li et al., 2000). In this study, it induced apoptosis *via* the mitochondrial pathway through decreasing the expression level of Bcl2 and Bcl-xL and simultaneously increasing the Bax expression level that in turn activates caspase-9 in HNC cell lines YCU-N861 and HPV18 transformant YCU-H891 cell line. Treatment with EGCG inhibited the phosphorylation of EGFR, STAT3, and ERK proteins. It also inhibited the basal and transforming growth factor α-stimulated c-fos and cyclin D1 promoter activity. It decreased the level of cyclin D1 and pRB, accounting for the cellular arrest in the G1 phase (Masuda et al., 2001). The efficacy of the therapies used for the treatment of HNC can be enhanced by the incorporation of EGCG in current therapeutic regimens. Currently, anti-EGFR antibodies or specific tyrosine kinase inhibitors are being used in combination with radiation and certain chemotherapy agents in clinical trials for various types of cancer, as inhibition of the EGFR-related signal transduction pathway enhances the cytotoxic effects of radiation or various chemotherapy agents (Wu et al., 1995; Dent et al., 1999; Bonner et al., 2000). Hence, EGCG may have certain advantages over EGFR antibodies or selected tyrosine kinase inhibitors, as it is relatively inexpensive, natural, and nontoxic, and hence might be useful in administering for a longer period without any adverse effects. Clinical efficacy of EGCG still needs to be determined, and the direct correlation between chemopreventive effect of EGCG and HPV activity is yet to be established by further *in vitro* and *in vivo* studies. Also, the p53 status during EGCG administration needs to be determined as 50% of HNC carry mutations in the p53 gene, which in turn can modulate effects of EGCG (Wang et al., 2020).

**TABLE 9:** Chemotherapeutic and Chemopreventive phytochemicals/Herbal derivative with anti-cancer and anti-HPV activity in HNC.

Phytochemicals/ Herbal Products; PubChem-CID (Class)	Cell type/Model/Clinical	Stage of Cancer	Anti-cancer activity in HPV positive cell	HPV specific effect	Ref.
EGCG (Green Tea Polyphenol (-)-Epigallocatechin-3-gallate)	*In vitro* study: **YCU-N861** ^a^ (NPSCC) and **YCU-H891#** (Hypopharyngeal SCC)	Promotion	• G1 phase Cells ↑; Cyclin D1 ↓, pRB ↓	NA	Masuda et al. (2001)
			• Apoptosis ↑; Bcl2 ↓, Bax ↑, Caspace 9↑: apoptosis↑.		
CID: 65064			• Phosphorylation of EGFR, STAT3, ERK proteins ↓		
			• Basal and transforming growth factor α-stimulated C-fos and Cyclin D1 promoter activity ↓		
Emodin *(Polygonum multiflorum)*	*In vitro* study: **CNE-1#** (NPSCC epithelioid cell line)	Promotion and progression	• ROS ↑; Oxidative damage ↑	NA	Hou et al. (2013)
			• Apoptosis ↑		
CID: 3220 (Trihydroxyanthraquinone)			• HIF-1α ↓		
			• Promotion of survival ↓		
Curcumin	*In vitro* study: **93VU147T** ^a^ (OSCC)	Promotion and progression	• Cell viability ↓	• HPV16/E6 mRNA ↓	Mishra et al. (2015)
CID: 969516 (Phytopolylphenol)			• Bcl-2 ↓, cIAP2 ↓, Bax ↑	• E6-mediated p53↓	
			• AP-1 ↓, NF-KB ↓		
CIP-36 (Novel podophyllotoxin derivative)	*In-vitro* study: **KB** ^a^ (HeLa contaminant epidermal carcinoma of mouth)	Promotion and Progression	• Cell proliferation ↓	NA	Cao et al. (2015)
			• Blocks cells in S/G2+M phase		
			• Effects DNA cleavage mediated by Human Topo IIα		
Sulforaphan	*In-vitro* study: **UM-SCC-22A** (Hypopharyngeal SCC), **UM-SCC-1** (OSCC), **CAL33** (Tongue OSCC), and **UPCI: SCC090** ^a^ (Tongue OSCC).	Initiation, promotion and progression	• NRF2 signaling ↑	NA	Bauman et al. (2016)
CID: 5350 (Isothiocyanate	*In-vivo* study: Female **C57BL/6 mice** (5–6 weeks; 18 mice/group).		• STAT3 phosphorylation ↓		
	Clinical study: 10 human subjects		• Promotes cell death		
			• Consumption of BSE (Broccoli sprout extracts rich in sulforaphane) beverage demonstrated NRF2 pathway activation in oral mucosa.		
6-Gingerol	*In-vitro* study: **KB** ^a^ (HeLa contaminant epidermal carcinoma of mouth), **SCC4** (Tongue SCC).	Promotion and progression	• Tumor cell proliferation ↓	NA	Kapoor et al. (2016)
CID: 442793 (Beta-hydroxy ketone)					
			• Cytotoxicity ↑		
			• Caspase-3 pro-apoptotic activity ↑		
			• Sub-G1 cells ↑; G2 and S phase arrest		
Curcumin	*In-vitro* study: **UD-SCC-2** ^a^ (Hypopharyngeal SCC); **UPCI:SCC131** (OSCC), **UPCI:SCC84** (OSCC)	Promotion, Proliferation and progression	• Cell growth↓	• HPV 16 E6/E7 ↓	Bano et al. (2018)
CID: 969516 (Phytopolylphenol)			• Orosphere formation ability↓	• miRNA21 ↓	
			• Cytotoxic effect		
Thymol	*In-vitro* study: **CAL27** (Tongue OSCC), **SCC4** (Tongue OOS), **SCC9** (Tongue OSCC)	Initiation, promotion and progression	• Cellular proliferation↓; Apoptosis↑	NA	De La Chapa et al. (2018)
CID: 6989 (Monoterpene)			• Long lasting cytotoxic effects		
	*In-vivo* study: athymic nude mice, **Cal27- and HeLa-derived** ^a^ mouse xenografts.		• Long lasting cytotoxic effects		
			• TRPA1 activation		
			• Mitochondrial membrane potential↓		
			• Mitochondrial dysfunction		
			• *In vivo* Tumor growth↓		
Apigenin (40 ,5,7, -trihydroxyflavone, AP)	*In-vitro* study: **SHEE** ^a^ ^,^ ^b^ (Human esophageal epithelial cell line)	Promotion and progression	• Migration Ability↓	NA	Yin et al. (2020)
			• G1 phase arrest; CDK4↓, Cyclin-D1↓, pRB↓		
			• Apoptosis↑ activation of caspase-3.		
			• DNA Alkylation↓		

a HPV16/18 positive cell lines **NA**: No HPV-specific effects have been reported.

b Some research papers have classified Human esophageal carcinoma under Head and neck squamous cell carcinoma but it should be classified under the broader category of digestive system carcinoma.

**Abbreviations:** AP-1: Activator protein 1; Bax: Bcl-2-associated X protein; Bcl2: B-cell lymphoma 2; BSE: Broccoli Sprot extracts; CDK: Cyclin-dependent kinases; cIAP: Calf Intestinal Alkaline phosphatase; EGFR: Epidermal growth factor receptor; ERK: Extracellular-signal-regulated kinase; HIF-1α: Hypoxia-inducible factor 1-alpha; HPV: Human papillomavirus; IFN-γ: Interferon gamma; NF-Kβ: Nuclear factor kappa light chain enhancer of activated B cells; NRF2: Nuclear factor erythroid 2-related factor 2; pRB: phosphorylated Retinoblastoma; STAT3: Signal transducer and activator of transcription 3; Topo IIα: topoisomerase II- alpha; TRPA1: Transient receptor potential cation channel, subfamily A, member 1.

Emodin, a natural trihydroxyanthraquinone, has lower oxidation–reduction potential than that of oxygen; hence, under hypoxic conditions, it can be reduced to cytotoxic agent, sensitizing the cells to irradiation (Zhu et al., 2005; Zou et al., 2010; Schwartz et al., 2011). It affected the NPC cell (CNE1, a HeLa contaminated cell line) promotion and progression by inducing oxidative damage by significantly increasing the expression level of ROS, which induces apoptosis and downregulates mRNA and protein levels of HIF-1α. It also reduces the promotion of survival of carcinoma cells and induces cell cycle arrest at the G2/M phase. Hence, exposure of NPC cells *in vitro* and xenografts *in vivo* to emodin enhanced their radiosensitivity (Hou et al., 2013). Therefore, incorporation of emodin, a bioreductive agent, represents a viable therapeutic strategy targeting HIF-1α, by enhancing cytotoxicity of chemotherapeutic drugs *via* modulation of redox status of cancer cells and multidrug resistance reversal (Yi et al., 2004; Brown et al., 2007; Cai et al., 2008; Huang et al., 2008). It may also serve as an effective radiosensor, thereby improving efficacy of radiation therapy in radiation-resistant cancer cells. Moreover, since emodin can effectively enhance the radiosensitivity *in vivo*, it holds a potential as a radiosensitizing drug for NPC patients in future. Still a direct correlation between emodin and HPV-activity needs to be established.

Curcumin (diferulolylmethane), an active component of turmeric and a perennial herb, has been shown to suppress the expression of HPV oncogenes mediated by downregulation and reduced transactivation of AP-1 and NF-kB superfamily members, representing a novel mechanism regulating HPV-induced oral carcinogenesis (Li et al., 1993; Prusty and Das, 2005). Its effect was also observed in HPV-positive 93VU147T cells. The cell viability is reduced significantly. It also induces apoptosis by decreasing the expression level of antiapoptotic factors such as Bcl-2 and cIAP2, and inducing proapoptotic factors like Bax. It downregulated the protein expression of AP-1 members: c-Jun, JunD, and JunB along with NF-kB members, p50 and p65. Curcumin also tends to show HPV-specific effects like reducing the mRNA levels of HPV16/E6, which in turn inhibits E6-mediated p53 degradation. Hence, curcumin exhibits therapeutic potential for HPV16-mediated oral oncogenesis suppression (Mishra et al., 2015). Similar result was observed in a later study on curcumin as phytochemical having both anti-HNSCC and anti-HPV activities, which was carried out on UD-SCC-2, UPCI:SCC131, and UPCI:SCC84 cell lines. It affected cancer promotion, cellular proliferation, and progression. Curcumin inhibited cancer cell growth and orosphere formation ability. Also, it induced cytotoxic effect along with HPV-specific effects like decreasing the expression level of HPV16 E6/E7 oncoproteins, and downregulated mi-RNA21 expression significantly in HPV-positive oral CSCs. Hence, curcumin can sensitize the HPV-positive oral CSCs, thus making the cancer treatment more effective when used in combination with standard anticancer drugs or radiation, depicting its potential as a therapeutic agent. Further studies are required for deciphering the therapeutic effects of curcumin by determining its solubility and bioavailability, mechanism(s) of action, and potential molecular targets (Bano et al., 2018).

Sulforaphane, an isothiocyanate, derived from broccoli sprout extracts; treatment of HPV-negative HNC cell lines–UM-SCC-22A, UM-SSC-1, and CAL33–and HPV-positive cell line SSC090 led to dose- and time-dependent stimulation of NRF2 signaling for carcinogen detoxication. It also dephosphorylated inhibitedSTAT3 and promoted cell death. Similar effects were also observed in *in vivo* and clinical study including female C57BL/6 mice (5–6 weeks; 18 mice/group) and 10 human subjects, respectively. The pilot clinical trial demonstrated consistent bioavailability of sulforaphane, promising sustainable chronic administration. Although it is a cost-effective and natural product, further studies planned with encapsulated broccoli extract are required to enhance the ease of acceptability and dispensing. Also, HPV-specific chemopreventive effects are yet to be determined (Bauman et al., 2016).

6-Gingerol, a β-hydroxy ketone, derived from ginger rhizome, inhibited tumor cell proliferation and induces cellular toxicity, cell cycle arrest, apoptosis, and caspase 3/7 activation, as observed in KB and SCC4 cells. Also, the caspase-3–dependent proapoptotic activity was stimulated. It also inhibited cell cycle progression arresting the cells in G2 and M phases. Hence, 6-gingerol can be considered as a safe and potent chemotherapeutic/chemopreventive compound acting *via* cell cycle arrest and induction of apoptosis (Kapoor et al., 2016). Further studies should be directed toward determination of the chemopreventive effects of 6-gingerol in *in vivo* conditions and clinical trials along with direct correlation with HPV activity.

Thymol, a monoterpene derivative phenol, is a TRPA1 agonist found in thyme and oregano. It inhibited cellular proliferation and exhibited long-lasting cytotoxic effects as observed in CAL27, SSC4, and SSC9 cell lines. It also inhibited tumor growth *in vivo* as observed in CAL27 and HeLa-derived mouse xenografts. It induces the activation of TRPA1 and apoptosis *via* the mitochondria-dependent pathway. It promoted mitochondrial dysfunction *via* reducing mitochondrial membrane potential significantly (De La Chapa et al., 2018). Its HPV-specific effects still need to be determined along with the determination of bioavailability and tolerability to understand its therapeutic effects for future incorporation into cancer treatment.

Apigenin, a flavonoid, found abundantly in flowers of plants, vegetables, and fruits, exerts anticarcinogenic effects *via* preventing malignant transformation of cells, regulating cell signal transduction pathways, increasing apoptosis, and modulating cell cycle (Fang et al., 2007; Zhao et al., 2011; Zhu et al., 2013; Salmani et al., 2017; Yang et al., 2018a). It inhibited cancerous cell migration ability and arrested them in the G1 phase as observed in SHEE cells induced by HPV-18 and 4-(methylnitrosamino)-1-(3-pyridyl)-1-butanone (NNK). It downregulated the expression of CDK4, cyclin D1, and pRB, affecting cell cycle. Apigenin also induced cellular apoptosis *via* caspase-3 activation and inhibits DNA alkylation. With low toxicity and various beneficial bioactivities, apigenin can be considered as a potential chemopreventive agent against cancers, particularly, in smokers with HR-HPV coinfection (Yin et al., 2020).

Hence, most of the phytochemicals mentioned above showed anticancer activity in HPV-positive cells, where only a limited studies focused on HPV-specific effects. Thus, considerable attention should be paid to analyze the correlation between anti-HNC and anti-HPV activity of the phytochemicals as a chemopreventive and chemotherapeutic measure to prevent HPV-HNC.

### Critical Issues Associated With the Use of Phytochemicals

Despite their encouraging pharmacological activities, there are bottlenecks in the translation of phytochemical-based therapies applicable in clinical settings.

***Low bioavailability***: Many phytochemicals suffer from having poor aqueous solubility and low retention in blood circulation. Pharmacological concentration of these phytochemicals in blood and tumor tissues is low because of poor absorption, high rate of metabolism, chemical degradation, and speedy clearance. It has been reported that serum levels of curcumin were quite low, reaching a maximum of 0.06 ± 0.01 μg/ml after oral administration of 500 mg/kg in rats (Yang et al., 2007). Ravindranath and Chandrasekhara (1980) also demonstrated that 40% of curcumin gets excreted unchanged in feces when orally administered to rats (Ravindranath and Chandrasekhara, 1980). A pilot study conducted among 10 healthy patients also reported poor bioavailability of sulforaphane with a regimen of topical exposure to sulforaphane-rich broccoli sprout extracts (Bauman et al., 2016). Chen et al. (1997) investigated the plasma pharmacokinetics of EGCG in rats and found the oral bioavailability of only 1.6% after a 75 mg/kg oral dose and a 10 mg/kg intravenous dose (Chen et al., 1997). Similarly, circulation half-life of resveratrol when administered through i. v. was few minutes and showed rapid elimination (Marier et al., 2002), whereas EGCG and quercetin attain low concentrations in blood, which is inadequate for antitumor activity (Lagoa et al., 2017).

Obstacles associated with the use of phytochemicals for treating and preventing cancer can be overcome with advances in the field of nanotechnology. A 10-fold dose advantage was achieved without any loss of effectiveness by encapsulating ECGC in polylactic acid–polyethylene glycol nanoparticles (Siddiqui et al., 2009). Increased absorption was also reported by nanoparticle encapsulation of curcumin despite its low solubility in water. Additionally, curcumin loaded poly lactic-co-glycolic acid nanoparticles increased the oral bioavailability to nine times that of the native form, with piperine as absorption enhancer (Shaikh et al., 2009). Further advancements in this field should be encouraged.

***Toxicity***: Although phytochemicals may show toxicity when administered in high doses, they exhibit less adverse effects than conventional therapies. In a clinical trial with 50 oral leucoplakia patients, significant toxicity, severe enough to cause withdrawal of 6 patients, was observed with the use of isoretinoin (Garewal et al., 1999). Additionally, not all phytochemicals are safe for consumption. It has been found that a few natural compounds such as capsaicin (chilli pepper), cycasin, and cycas seed are tumor-promoting and must be avoided (Bode and Dong, 2015). Moreover, unregulated use of phytochemicals may have a danger of contamination by potential carcinogens.

***Pharmaceutical industry challenges***: Pharma-research into phytochemicals and herbal derivatives has experienced a slow decline during the recent times (Koehn and Carter, 2005; Katiyar et al., 2012). This can be attributed to advancements in high-throughput screening technology against defined molecular targets, advances in genomics, molecular and cellular biology, development of combinatorial chemistry, and a declining importance among large pharma-companies on the commercial considerations of phytochemicals that are often associated with poor financial returns and nearly absent IPR protection. Unique features of natural compounds such as a greater number of chiral centers, higher number of oxygen atoms, and greater molecular rigidity pose further challenges for medicinal chemists as they develop analogs to reduce toxicity, improve absorption, or to improve the efficacy, which is often achieved by adding or deleting selected functional groups.

***Poor independent agents***: While phytochemicals may not be efficient as standalone chemotherapeutic agents, many groups have established their efficacy as adjuvants to traditional therapies. A study demonstrated the benefits of combining sulforaphane with cisplatin and 5-fluorouracil (Elkashty et al., 2018). Sulforaphane increased the cytotoxicity of cisplatin and 5-fluorouracil by two-fold and ten-fold, respectively. It did not alter the viability and functions of noncancerous stem cells. Sulforaphane combined treatments successfully inhibited cancer stem cell colony formation, sphere formation, and tumor progression *in vivo*. In an Italian study conducted among 23 patients undergoing treatment with 5-fluorouracil and cisplatin, prolonged responses were reported with the use of retinol palmitate in chemotherapy intervals. Toxicity levels were acceptable, and treatment did not interfere with the quality of life (Recchia et al., 1993). A study also observed significant growth inhibition and enhanced apoptosis in HNC cells with the use of curcumin along with 5-fluorouracil or doxorubicin. The study thus demonstrated the significant potential of combining curcumin with 5-fluorouracil or doxorubicin as a treatment modality for HNC management (Sivanantham et al., 2016).

***Preclinical efficacy vs. clinical response***: The cause for discrepancy in effectiveness of phytochemical agents in preclinical and human clinical trials has been conjectured to arise because of differences in dosage, metabolic differences, bioavailability, differences in circulating tissue levels of chemopreventive agents in humans and animals, exposure conditions to damaged tissue vs normal tissue, follow-up time, and the assessed ends. Second, high doses are often administered to animals in contrast to low doses admisible to humans in clinical trials. Although animal models have significantly helped in the identification of carcinogens, and chemopreventive and chemotherapeutic agents, they are not available for every HNC organ site. Furthermore, existing models cannot mimic human exposure complexities of carcinogens, metabolic competence, turnover of cells, and their repair capacity.

## Conclusion and Future Prospective

Phytochemicals show immense potential in the field of HNC chemotherapy and chemoprevention agents. In this evolving landscape, the success of employability of phytochemicals depends on our ability to decipher their molecular mechanics. Using phytochemicals in combination with another or in conjunction with existing chemotherapeutic practices or an alternate therapy is an area worth exploring.

**FIGURE 4:**
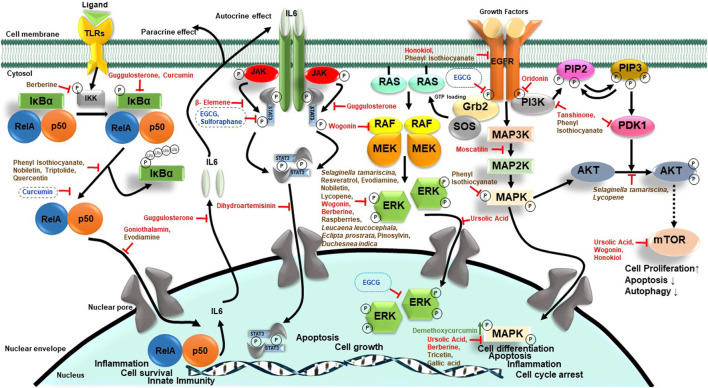
Effect of phytochemicals on different oncogenic signaling pathways of HNC. Summarized here are chemotherapeutic (**red**) and chemopreventive (**brown**) phytochemicals that target different signaling pathways along with specific phytochemicals (**blue**) demonstrating anti-HPV activity in HPV-positive HNC.

We have also observed that there has not been much phytochemical-related research on HPV-induced HNC. However, numerous phytochemicals that are effective against HPV-induced cervical cancer have been reported in the literature (Bharti et al., 2018). In today’s era, therapies to distinguish HPV-positive HNC from HPV-negative HNC are required. As HPV-positive HNC has better outcomes, the tumors can be treated with well-established phytochemicals targeting the HPV-mediated carcinogenic mechanisms. Thus, it might be valuable to study whether these phytochemicals can find application in HNC treatment and prevention. Activity of these phytochemicals can be checked on HNC cell lines or *in vivo* in laboratory conditions and can also be screened by using bioinformatic tools. There is a strong requirement to develop HPV-based concurrent therapies so that HNC can be treated more effectively. There are many associated challenges with the use of natural compounds. In pharmacological doses, the adverse effects of these natural compounds such as increased toxicity and low bioavailability are amplified. For chemoprevention to be feasible in treating premalignant lesions, the compound must be well tolerated and have long-lasting benefit. Moreover, the various signaling pathways contributing to HNC tumorigenesis mandate the use of compounds with multiple molecular targets. It is noteworthy that molecular targets of many such phytochemicals in active HNC are now well known (Figure 4). It is also worthy to note that not many clinical studies have been conducted despite discovery of numerous phytochemicals with multiple molecular targets. In order to determine the safety and efficacy of phytochemicals, it is imperative that more of such clinical studies, with different phytochemicals, are funded and conducted. Challenges associated with the use of phytochemicals such as low bioavailibilty, and toxicity can be possibly overcome with the use of chemical analogs, adjuvant therapies, and nanoparticle delivery mechanisms. Hence, a number of studies on phytochemicals against HPV-driven HNC are now accumulating; a comparative account on their relative efficacy is needed and should be addressed to harness the potential of phytochemicals in clinical studies. Research in these areas needs encouragement for effective management of HPV-positive HNC in future.

## Glossary

4NQO: 4-nitroquinoline 1-oxide
5-FU: 5-fluorouracil
Akt: protein kinase B
AP-1: activator protein 1
Bax: Bcl-2–associated X protein
Bcl-2: B-cell lymphoma 2
Bcl-xL: Bcl-2 homolog B-cell lymphoma-extra large
BH3: Bcl-2 homology 3
Bim: Bcl-2–interacting mediator of cell death
BRB: black raspberry
CAPE: caffeic acid phenethyl ester
Cdc2: cell division control 2
CDK2/4: cyclin-dependent kinase 2/4
CDKN2A: cyclin-dependent kinase inhibitor 2A
c-Fos: cellular oncogene Fos
CIAP2: calf intestinal alkaline phosphatase
Cip1: CDK interacting protein 1
c-Jun: cellular Jun
COX-2: cyclooxygenase-2
c-Raf: c-Rapidly accelerated fibrosarcoma
CT: concurrent chemotherapy
E6/7: Early protein 6/7
EBV: Epstein–Barr virus
ECGC: (-)-Epigallocatechin-3-gallate
EGFR: epidermal growth factor receptor
ERK: extracellular signal–regulated kinase
FOXO1: forkhead box protein O1
Fra-2: Fos-related antigen 2
GSK3: glycogen synthase kinase 3
SK3β: glycogen synthase kinase 3 beta
HN: head and neck
HER2: human epidermal growth factor receptor 2
HIF-1 α: hypoxia-inducible factor-1 α
HNC: head and neck cancer
HNSCC: head and neck squamous cell carcinoma
HO1: heme oxygenase 1
HPV: human papillomavirus
IARC: international agency for research on cancer
IC50: half maximal inhibitory concentration
IgE: immunoglobulin E
IKK: inhibitor of nuclear factor-κB kinase
IL-6: interleukin 6
iNOS: inducible nitric oxide synthase
IRS-1: insulin receptor substrate 1
IκB: inhibitor of nuclear factor-κB
IκBα: I-kappa-B-alpha
JAK2: janus kinase2
JNK: c-Jun N-terminal kinase
Kip2: kinase inhibitor 1
LC3: microtubule-associated protein 1A/1B-light chain 3
LCN2: lipocalin-2
LSCC: laryngeal squamous cell carcinoma
M1 type macrophages: activated macrophages
MAPK: mitogen-activated protein kinase
MDM2: mouse double minute 2 homolog
miR: micro RNA
MLKL: mixed lineage kinase domain-like pseudokinase
MMPs: metalloproteinases
mTOR: mammalian target of rapamycin
mTORC2: mTOR Complex 2
NF-κB: nuclear factor kappa light chain enhancer of activated B cells
NPC: nasopharyngeal carcinoma
NPSCC: nasopharyngeal squamous cell carcinoma
OED: oral epithelial dysplasia
OPC: oropharyngeal carcinoma
OPMDs: oral potentially malignant disorders
OPSCC: oropharyngeal squamous cell carcinoma
OSCC: oral squamous cell carcinoma
P70S6K: 70-kDa ribosomal protein S6 kinase
PCNA: proliferating cell nuclear antigen
PD1: programmed cell death protein 1
PI3K: phosphatidylinositol 3-kinase
PIP2: phosphatidylinositol-4,5-bisphosphate
PIP3: phosphatidylinositol-3,4,5-bisphosphate
PKA-AMPK: protein kinase A–AMP-activated protein kinase
PPARδ: peroxisome proliferator–activated receptor delta
pRB: phosphorylated retinoblastoma protein
PRR5: proline rich protein 5
pSTAT3: phosphorylated signal transducer and activator of transcription 3
PTEN: phosphatase and tensin homolog
Raf: rapidly accelerated fibrosarcoma
RBP: RNA-binding protein
RICTOR: rapamycin-insensitive companion of mammalian target of rapamycin
RIPK1: receptor-interacting serine/threonine-protein kinase 1
ROS: reactive oxygen species
RT: radiation therapy
RTK: receptor tyrosine kinase
S6K1: ribosomal protein S6 kinase beta-1
SCC: squamous cell carcinoma
Sin1: stress-activated protein kinase-interacting protein
SLT: smokeless tobacco
SOX2: SRY (sex determining region Y)-box 2
STAT3: signal transducer and activator of transcription 3
TFs: transcription factors
TIMPs: tissue inhibitor of metalloproteinases
TNFα: tumor necrosis factor alpha
Topo IIα: topoisomerase II- alpha
TRPA1: transient receptor potential ankyrin 1
VEGF: vascular endothelial growth factor
WHO: World Health Organization
XIAP: X-linked inhibitor of apoptosis protein
ZSP: ZengShengPing
